# Supplementary material for: Lysosomal cystine export regulates mTORC1 signaling to guide kidney epithelial cell fate specialization
Source: Nat Commun. 2023 Jul 14;14:3994. doi: 10.1038/s41467-023-39261-3 (PMC10349091; doi:10.1038/s41467-023-39261-3)
Supplement: Supplementary file 1 — Supplementary Information [file 41467_2023_39261_MOESM1_ESM.pdf]

Lysosomal cystine export regulates mTORC1 signaling to guide kidney epithelial cell fate specialization

Marine Berquez et al.

Supplementary information

Supplementary Figures 1-14

Supplementary Tables 1-2

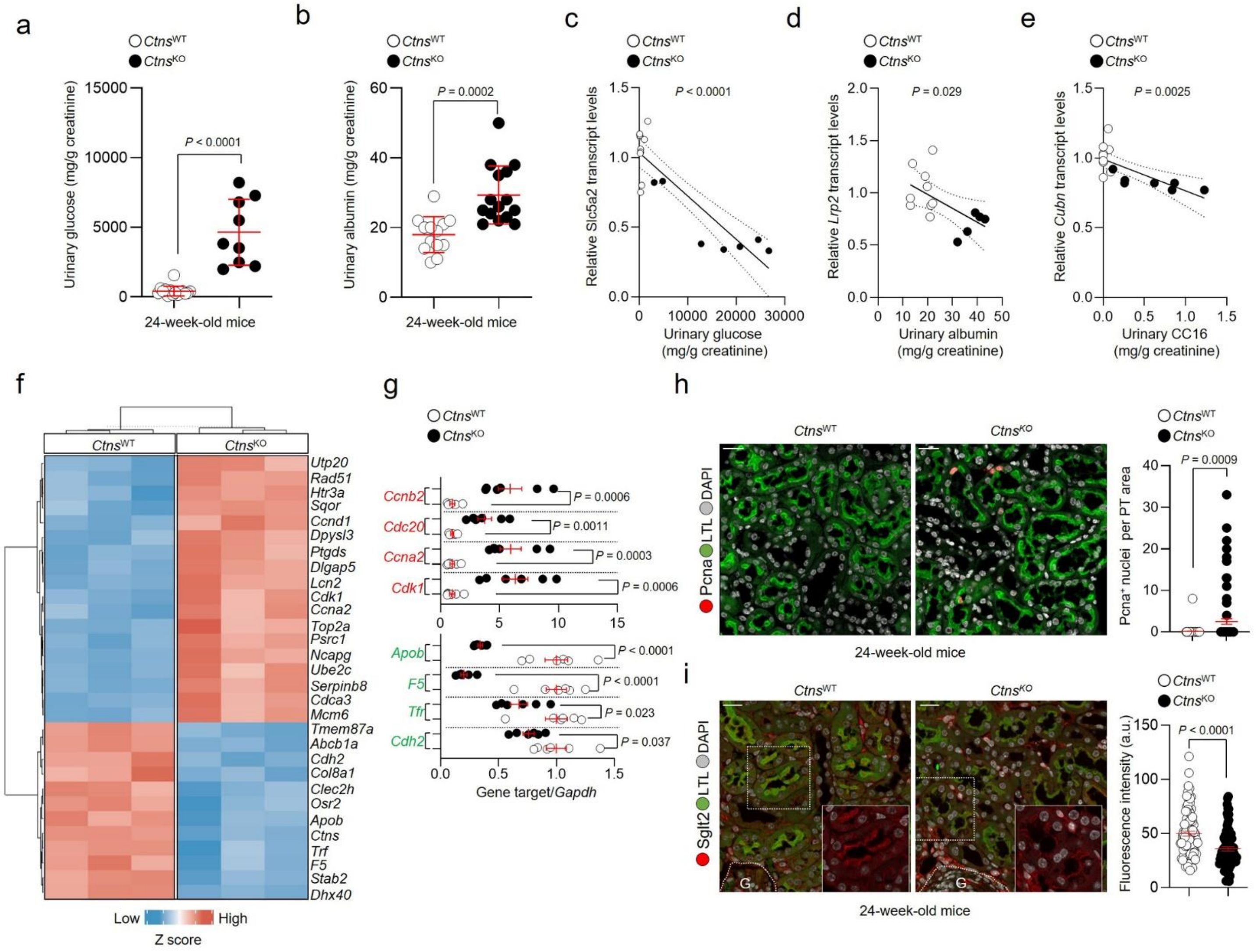

Supplementary Figure 1. Loss of differentiation and abnormal programs for growth and proliferation in the proximal tubule (PT) segments of CTNS-deficient mouse kidneys. (**a**) Glucose and (**b**) albumin levels in the urine samples of *Ctns* mice. For glucose: n=15 *Ctns*^{WT} and n=9 *Ctns*^{KO} mice and

for albumin: n=13 *Ctns*^{WT} and n=15 *Ctns*^{KO} mice, respectively. (**c**-**e**) Scatter plot of the correlation between the amount of glucose, albumin, and CC16/LMW proteins in the urine samples of *Ctns* mice and the relative transcript levels of (**c**) *Slc5a2*, (**d**) *Lrp2*, and (**e**) *Cubn*. The x axis is the gene expression, and the y axis is the abundance of solutes and proteins (expressed as mg/g creatinine) in the urine of experimental groups. Two-tailed *P*-value was calculated by Pearson's coefficient. For *Slc5a2*: n=10 *Ctns*^{WT} and n=7 *Ctns*^{KO} mice; and for *Lrp2*: n=9 *Ctns*^{WT} and n=5 *Ctns*^{KO} mice: and for *Cubn*: n=9 *Ctns*^{WT} and n=7 *Ctns*^{KO} mice, respectively. (**f**) Heatmap showing the expression of the top 30 differentially expressed genes (DEGs) in the PT segments obtained from 24-week-old *Ctns*-deficient mouse and (age-matched) control littermates; n=3 biologically independent animals in each experimental group. (**g**) Quantification of mRNA levels of the indicated genes; n=6 biologically independent animals in each experimental group. (**h** and **i**) Immunofluorescence staining and quantification of (**h**) the number of Pcna-positive nuclei (red) and (**i**) mean fluorescence intensity of Sglt2 signal in the LTL (Lotus Tetragonolobus Lectin)-positive PT segments (green) of *Ctns* mouse kidneys. For Pcna: n=86 *Ctns*^{WT} and n=90 *Ctns*^{KO} PT segments; and for Sglt2: n=61 *Ctns*^{WT} and *Ctns*^{KO} PT segments, both of which pooled from 3 biologically independent animals. Statistics calculated by unpaired two-tailed Student's *t* test. Plotted data represent mean ± SEM. Nuclei counterstained with DAPI (grey). Scale bars are 10 μm. Source data are provided as a Source Data file.

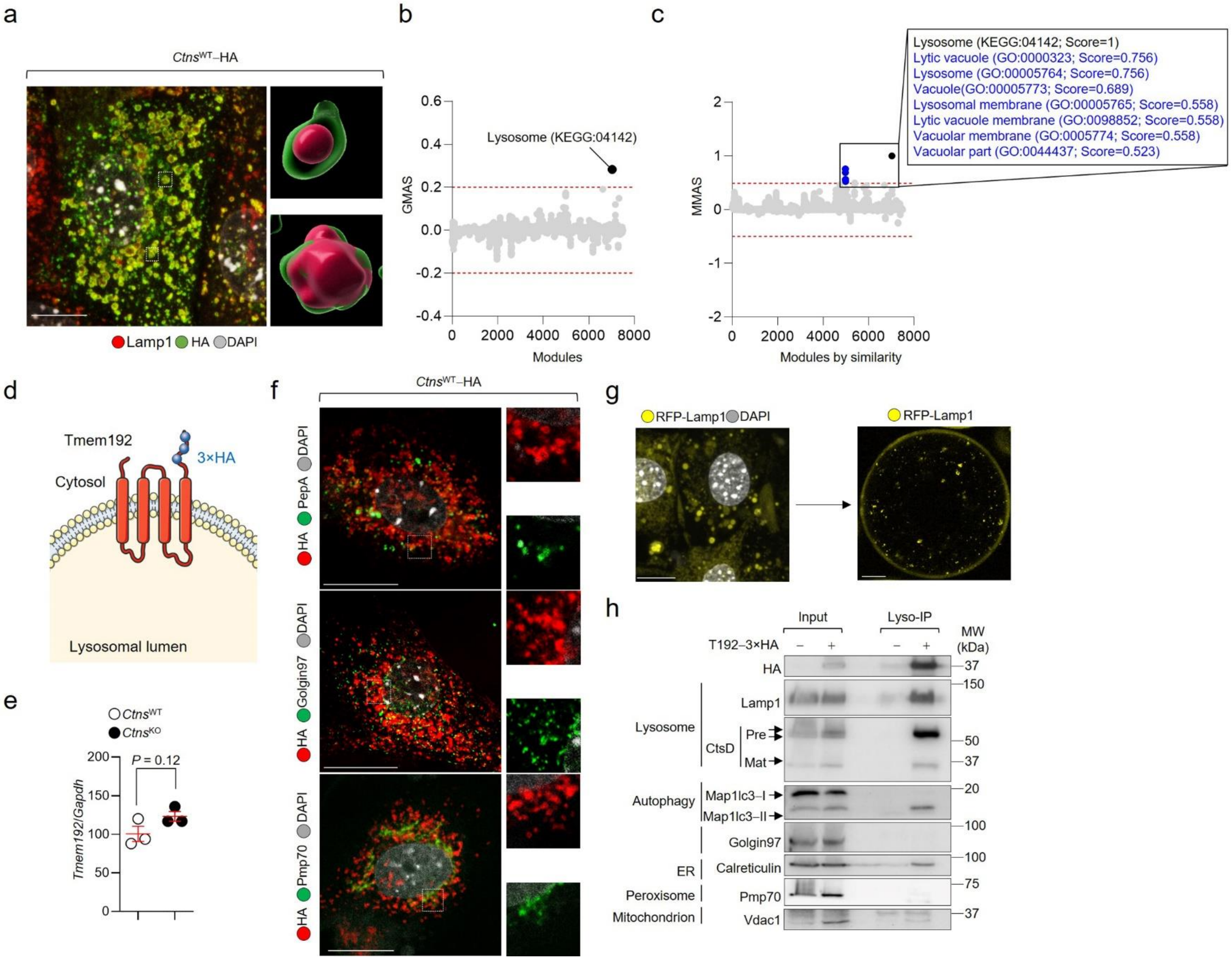
a
$Ctns^{WT}$–HA
Lamp1 HA DAPI
b
Lysosome (KEGG:04142)
GMAS
Modules
c
Lysosome (KEGG:04142; Score=1)
Lytic vacuole (GO:0000323; Score=0.756)
Lysosome (GO:00005764; Score=0.756)
Vacuole(GO:00005773; Score=0.689)
Lysosomal membrane (GO:00005765; Score=0.558)
Lytic vacuole membrane (GO:0098852; Score=0.558)
Vacuolar membrane (GO:0005774; Score=0.558)
Vacuolar part (GO:0044437; Score=0.523)
MMAS
Modules by similarity
d
Tmem192
3×HA
Cytosol
Lysosomal lumen
e
$Ctns^{WT}$
$Ctns^{KO}$
P = 0.12
Tmem192/Gapdh
f
$Ctns^{WT}$–HA
HA PepA DAPI
HA Golgin97 DAPI
HA Pmp70 DAPI
g
RFP-Lamp1 DAPI
RFP-Lamp1
h
Input
Lyso-IP
T192–3×HA
MW (kDa)
HA
Lamp1
Lysosome
CtsD
Pre
Mat
Autophagy
Map1lc3–I
Map1lc3–II
Golgin97
ER
Calreticulin
Peroxisome
Pmp70
Mitochondrion
Vdac1

Supplementary Figure 2. CTNS subcellularly localizes to the surface of the lysosome, and validation of the Lyso-IP workflow in the primary PT cells derived from *Ctns* mouse kidneys. (a) *Ctns*^{WT} mPTCs were transduced with adenoviral particles expressing hemagglutinin-tagged CTNS^{WT} (CTNS^{WT}-HA) for 2 days and immunostained for HA (green) and Lamp1 (red). Confocal microscopy and three-dimensional (3D) reconstruction of Z-stacked images of mPTCs confirmed the localization of CTNS at the surface of Lamp1-flagged lysosomes. (**b**-**c**) Systems biology-based toolkit (GeneBridge; https://systems-genetics.org) was exploited to uncover additional functions of *Ctns* – beyond its well-established role in the transport of cystine. Using large-scale mouse expression compendia, this computational pipeline enables the gene's function through its (anti)correlation with other genes for which the Gene Ontology (GO) terms/biological pathways that are already known. (**b**) Gene-module association analysis of *Ctns* and module-module association analysis of lysosome (KEGG:04142) module leveraging 252 and 269 mouse tissue datasets, respectively. Red dashed line indicates the threshold of significant association in **b** and **c**. (**d**) Schematic showing the localization of 3×haemagglutinin (HA)-fused transmembrane protein 192 (Tmem192×3HA) at the surface of the lysosome. Images were drawn using pictures from Servier Medical Art, which is licensed under a Creative Commons Attribution 3.0 Unported License (https://creativecommons.org/licenses/by/3.0/). (**e**) mRNA levels of Tmem192 in *Ctns* mPTCs were analyzed by RT-qPCR; n=3 biologically independent samples were analysed in each experimental group. (**f**) The cells expressing transiently Tmem192×3HA were pulsed with Bodipy FL-PepstatinA (1μM at 37°C for 1h; green) and immunostained for HA (red), or for Golgin-97 (green) and HA (red), or for Pmp70 (green) and HA (red) and analyzed by confocal microcopy. (**g**) Confocal images of (right panel) mPTCs transiently expressing Lamp1-RFP-3×Flag and (left panel) bead-purified lysosomes following immunoisolation method. (**h**) Immunoblotting for protein markers of various subcellular compartments in whole-cell lysates and purified lysosomes. Lysates were harvested from cells transiently expressing Tmem192×3HA; n=2 biologically independent experiments. Nuclei counterstained with DAPI (grey). Statistics calculated by unpaired two-tailed Student's *t* test. Plotted data represent mean ± SEM. Scale bars are 10mm in **a**, **f**, and **g**. Source data are provided as a Source Data file.

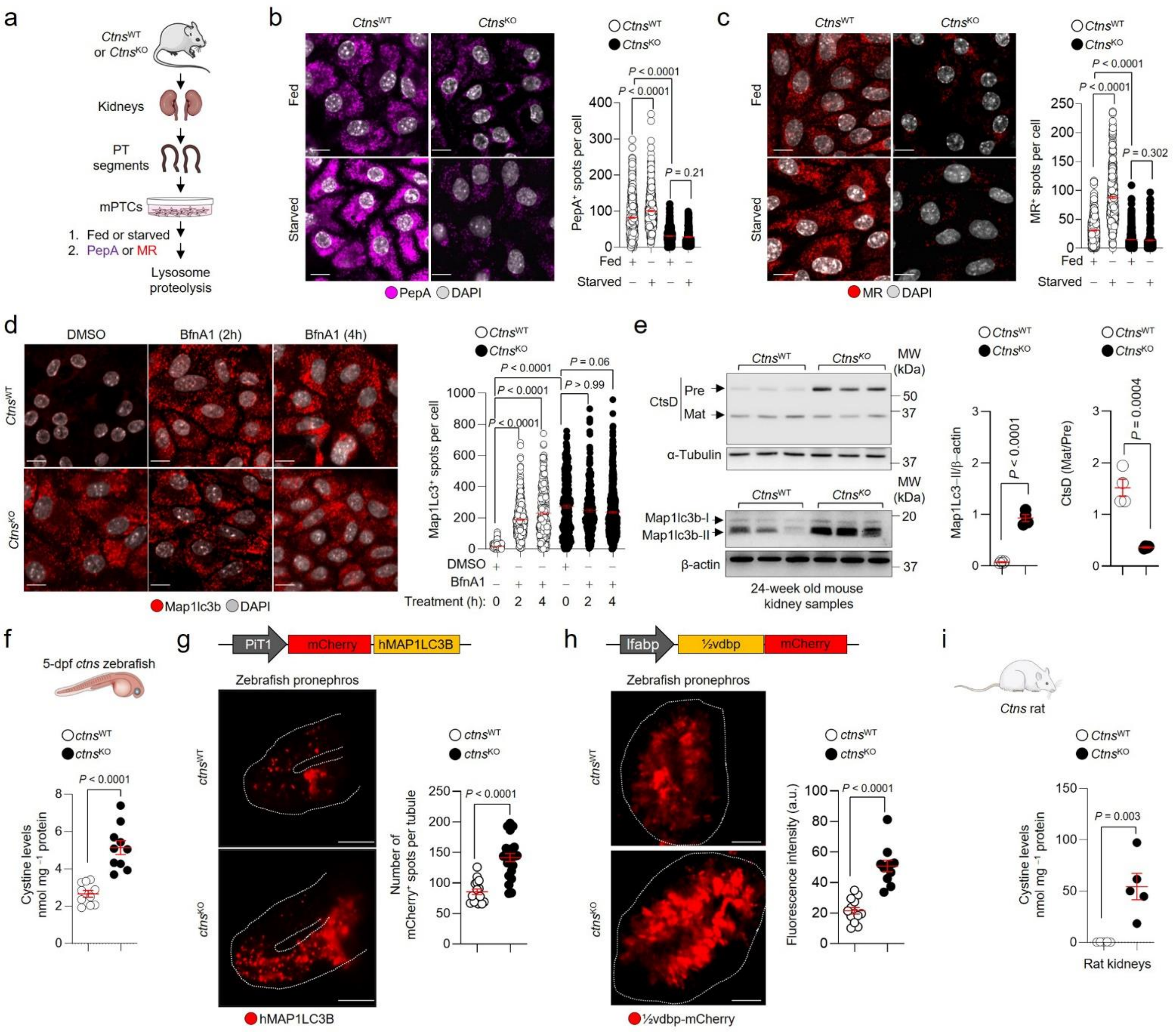
a
CtnsWT or CtnsKO
Kidneys
PT segments
mPTCs
1. Fed or starved
2. PepA or MR
Lysosome proteolysis
b
CtnsWT
CtnsKO
Fed
Starved
PepA DAPI
PepA+ spots per cell
P < 0.0001
P < 0.0001
P = 0.21
Fed
Starved
c
MR DAPI
MR+ spots per cell
P = 0.302
d
DMSO
BfnA1 (2h)
BfnA1 (4h)
Map1lc3b DAPI
Map1Lc3+ spots per cell
P = 0.06
P > 0.99
Treatment (h):
e
CtsD
Pre
Mat
α-Tubulin
Map1lc3b-I
Map1lc3b-II
β-actin
MW (kDa)
24-week old mouse kidney samples
Map1Lc3–II/β–actin
CtsD (Mat/Pre)
P = 0.0004
f
5-dpf ctns zebrafish
ctnsWT
ctnsKO
Cystine levels nmol mg −1 protein
g
PiT1
mCherry
hMAP1LC3B
Zebrafish pronephros
Number of mCherry+ spots per tubule
h
lfabp
½vdbp
Fluorescence intensity (a.u.)
½vdbp-mCherry
i
Ctns rat
P = 0.003
Rat kidneys

Supplementary Figure 3. Cystine storage disrupts the catabolic activities of the lysosomes in mPTCs, rat, and zebrafish lacking CTNS. (**a**) mPTCs were cultured under fed and starved conditions, pulsed with (**b**) Bodipy FL-PepA (PepA, 1μM at 37°C for 1h) or with (**c**) MagicRed Cathepsin B substrate (MR, 1μM at 37°C for 1h) and analysed by confocal microscopy. Representative confocal images and quantification of the number of PepA- and MR-positive structures per cell; For $PepA^{+}$ spots: n=579 fed *Ctns*^{WT} and n= 499 starved *Ctns*^{WT} cells; n= 691 fed *Ctns*^{KO} and n= 359 starved *Ctns*^{KO} cells; for MR^{+} spots: n=476 fed *Ctns*^{WT} cells and n= starved 495 *Ctns*^{WT} cells; n= 1006 fed *Ctns*^{KO} cells and n= 362 starved *Ctns*^{KO} cells, both of which pooled from 2 biologically independent experiments. (**d**) mPTCs were treated with non-saturating concentrations of BafilomycinA1 (BfnA1; 250nM for 4h) at the indicated times. The cells were immunostained for Map1Lc3b (red) and analyzed by confocal microscopy. Representative confocal images and quantification of the number of $Map1lc3b^{+}$ structures in *Ctns* mPTCs; n=422 (*Ctns*^{WT} + DMSO), n= 538 (*Ctns*^{WT} + BfnA1, 2h), and n= 418 (*Ctns*^{WT} + BfnA1, 4h) cells; and n=333 (*Ctns*^{KO} + DMSO), n= 289 (*Ctns*^{KO} + BfnA1, 2h), and n= 614 (*Ctns*^{KO} + BfnA1, 4h) cells, both of which pooled from 2 biologically independent experiments. (**e**) Immunoblot and quantification of the indicated proteins in the kidneys of *Ctns* mice; n=4 biologically independent animals in each experimental group. (**f** and **i**) Quantification of cystine levels by HPLC (**f**) in 5-dpf-*ctns* zebrafish larvae (n=10 biologically independent samples in each experimental group, with each dot representing a pool of 6 zebrafish) and (**i**) in 16-week-old Ctns rat kidneys (n=5 biologically independent animals in each group). (**g**) The *ctns* zebrafish were outcrossed with zebrafish expressing *PiT1::mCherry-hMAP1LC3B* (autophagosome marker, red) in the pronephros, and analysed by multiphoton microscopy. Representative images and quantification of the number of mCherry-positive structures in the pronephros of *ctns* zebrafish; n= 13 *ctns*^{WT} and n=11 *ctns*^{KO} zebrafish. (**h**) The *ctns* zebrafish were outcrossed with zebrafish expressing the ½vdbp-mCherry in the liver and analysed by multiphoton microscopy. Representative images and quantification of the mCherry fluorescence intensity in the pronephros of *ctns* zebrafish; n=16 *ctns*^{WT} and n=23 *ctns*^{KO} zebrafish. Plots represent mean ± SEM. Statistical calculated by unpaired two-tailed Student's *t* test in **e**, **f**, **g**, **h,** and **i**. Statistics calculated by Kruskal-Wallis followed by Dunn's multiple comparisons test in **b**, **c,** and **d**. Nuclei counterstained with DAPI (grey). Scale bars are10μm. Source data are provided as a Source Data file. Images in **a**, **f**, and **i** were drawn using pictures from Servier Medical Art, which is licensed under a Creative Commons Attribution 3.0 Unported License (https://creativecommons.org/licenses/by/3.0/).

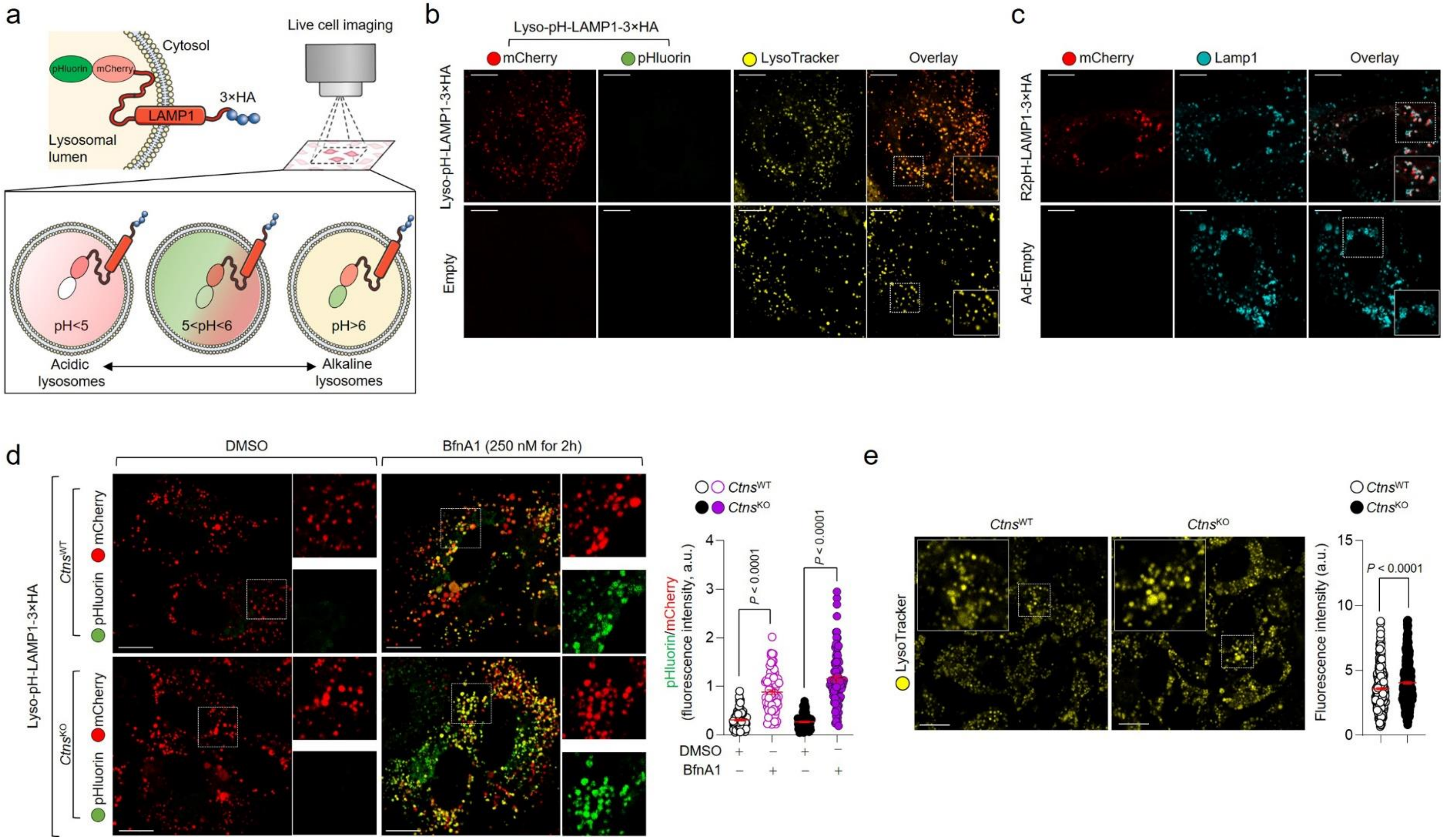

Supplementary Figure 4. Cystine storage does not affect the lysosomal acidification in mouse PT cells lacking CTNS. (**a**) Schematic showing the design and subcellular localization of a genetically encoded, bona fide biosensor that uses a pHluorin-mCherry linked to the luminal domain of the lysosome-localized protein, mouse Lamp1, with a cytosolic 3×HA tag [henceforth Lyso-pH-LAMP1-3×HA], to reliably measure the changes in pH within the lumen of the lysosome. When the lysosomes are properly acidified, the acidic environment triggers the quenching of the pHluorin signal, leaving only (acidic insensitive) red fluorescence. In an acidic lysosome, the biosensor has a low ratio value and is displayed in red, while it has a high ratio value in alkaline lysosomes and is displayed in yellow. Images were drawn using pictures from Servier Medical Art, which is licensed under a Creative Commons Attribution 3.0 Unported

License (https://creativecommons.org/licenses/by/3.0/). (**b**) *Ctns* mPTCs were transduced with an adenovirus that transiently expresses Lyso-pH-LAMP1-3×HA for 24 h and loaded with Lysotracker dye (1μM for 4 hours; yellow) or (**c**) fixed and immunostained for Lamp1 (cyan) and analyzed by confocal microscopy. These functional assays confirmed the expression of the pH biosensor at the surface of both LysoTracker- and Lamp1-positive lysosomes. All the experiments in **b** and **c** were performed two times independently, and all attempts were successful with similar results. (**d**) *Ctns* mPTCs expressing Lyso-pH-LAMP1-3×HA were treated with non-saturating concentrations of BafilomycinA1 (BfnA1; 250nM for 4h) and analyzed by confocal microscopy. Confocal images and quantification of green-to-red fluorescence intensity ratio in *Ctns* mPTCs; n=88 ($Ctns^{WT}$ + DMSO); n=82 ($Ctns^{WT}$ + BafnA1); n=104 ($Ctns^{KO}$ + DMSO); and n=91 ($Ctns^{KO}$ + BafnA1) cells pooled from 3 biologically independent experiments. (**e**) *Ctns* mPTCs were loaded with LysoTracker (1μM for 4 hours; yellow) and analysed by confocal microscopy. Representative confocal images and quantification of fluorescence intensity of LysoTracker signal in *Ctns* mPTCs; n=375 $Ctns^{WT}$ and n=372 $Ctns^{KO}$ cells pooled from 2 biologically independent experiments. Plots represent mean ± SEM. Statistics calculate by one-way ANOVA followed by Sidak's multiple comparisons test in **d**, and by unpaired two-tailed Student's *t* test in **e**. Scale bars are 10μm. Source data are provided as a Source Data file.

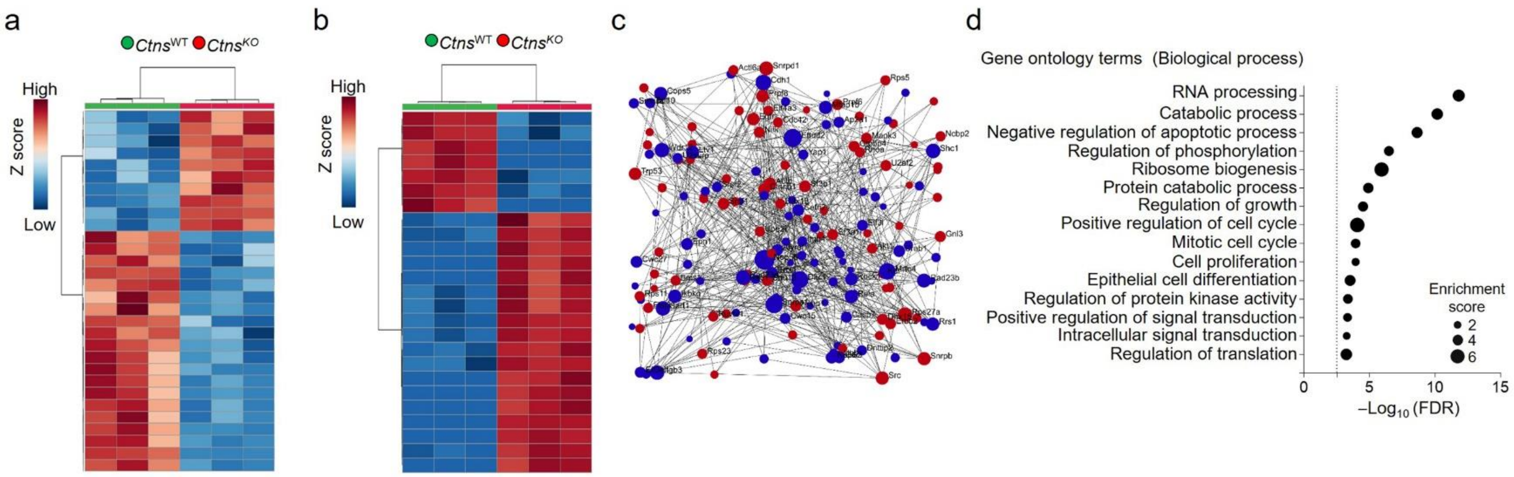

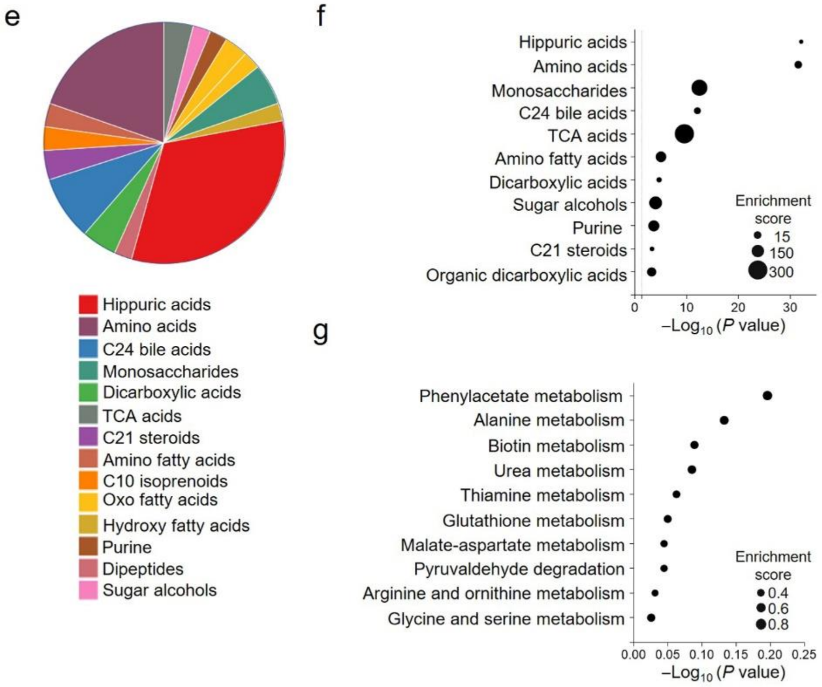

Set name	Metabolites	References
Amino acids	Betaine; Arginosuccinic acid; Glycine; Phenylalanine; L-Alanine; L-threonine; L-Isoleucine; L-Lysine; L-Cystine; Ornithine; Sarcosine; L-Arginine; L-Leucine; L-Homoserine; Ureidosuccinic acid; L-Valine; D-Alanine; Nicotinuric acid; D-Ornithine; D-Arginine; β–Leucine; L-Allothreonine; 5-L-Glutammyl-taurine; Norvaline; γ–Glutamyl–β–aminopropriononitrile	RefMet

Set name	Metabolites	References
Glutathione metabolism	Cysteinlglycine; Glycine; Glutathione; L–Glutamic acid; L–Alanine; NADP; NADPH; Pyroglutamic acid; Adenosine triphosphate; L-cysteine; γ–Glutamylcysteine; FAD; Zinc (II) ion; ADP; Water; Phosphoric acid; Hydrogen peroxide; Oxidized glutathione; γ–Glutamylalanine; Zinc; Bromomethane	SMPDB KEGG

Supplementary Figure 5. Proteome and metabolome profiling of the tubular cells lacking CTNS and accumulating cystine. (**a-b**) Heatmap showing the expression of the top 30 differentially expressed proteins (**a**) and produced metabolites (**b**) in *Ctns* mPTCs derived from 24-week-old mouse kidneys; n=3 biologically independent animals in each group. (**c**) Protein-protein interaction (PPI) map and (**d**) over-representation analysis of top biological processes (Gene Ontology, GO terms; Biological process) associated with differentially expressed proteins [$\log_2$FC < -0.5 and Log_2FC > 1, *P* value < 0.05] using the Network Analyst toolkit (www.networkanalyst.ca). This web-based biological network analysis project and visualize gene/proteins within their biological networks to explore their statistical and functional relationships. The computational tool embeds genes/proteins within biological networks from 15 different databases. These networks are mined to extract the genes/proteins, miRNA, drugs, chemicals, or diseases that have the strongest relationships to genes/proteins in the uploaded list (seed genes). Black dashed line indicates the threshold of significant enrichment. Black circles indicate the size of the enrichment score. *P* value was calculated from the enrichment analysis in NetworkAnalyst. (**e**) Pie charts showing the distribution of sub-chemical metabolite sets and (**f**) metabolite set and (**g**) pathway enrichment analyses of differentially produced metabolites [$\log_2$FC < -0.5 and Log_2FC > 1, *P* value < 0.05]. Black circles indicate the size of the enrichment score. *P*-values were calculated from the enrichment analysis in MetaboAnalyst.

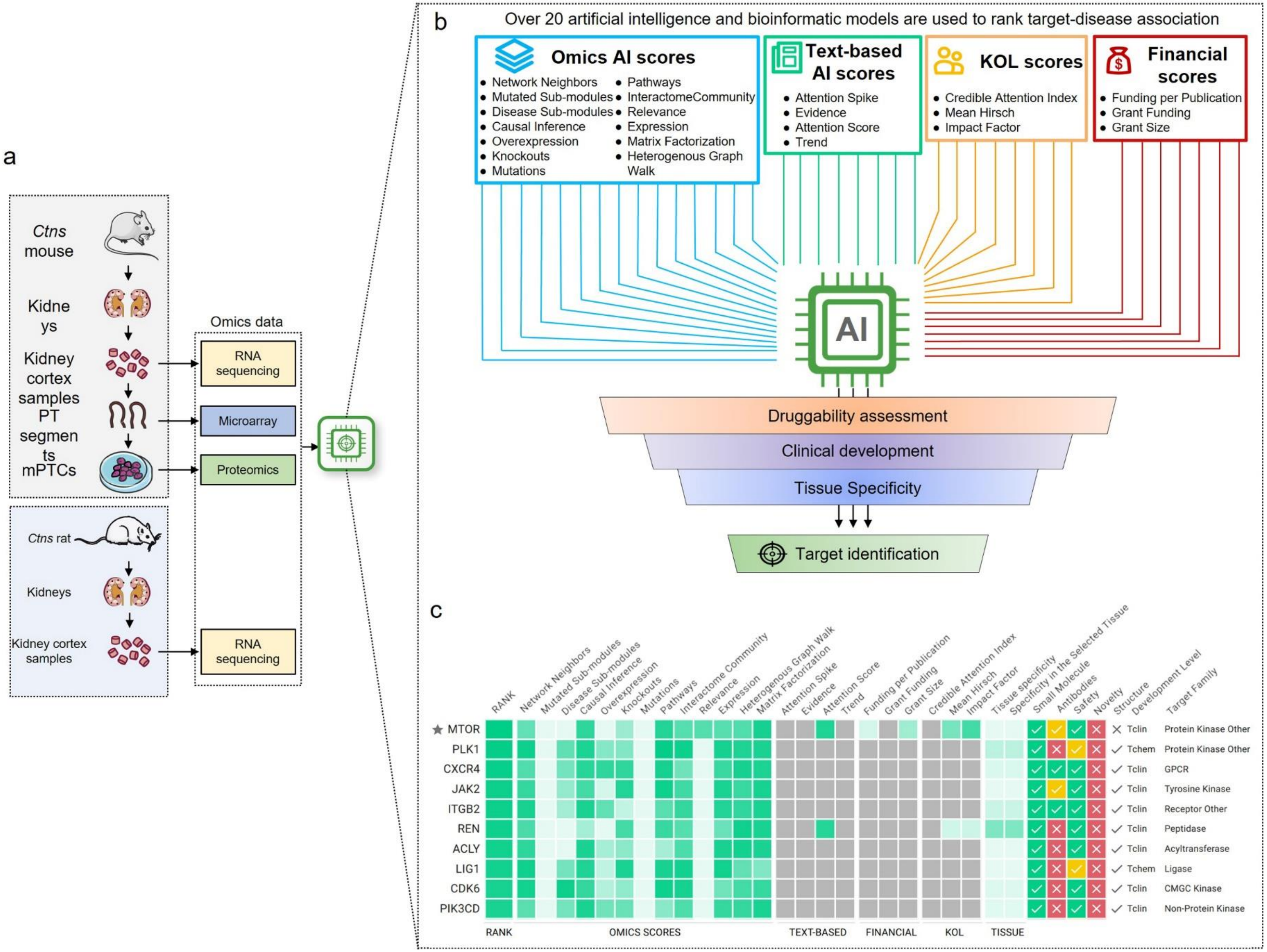

a
Ctns mouse
Kidneys
Kidney cortex samples
PT segments
mPTCs
Ctns rat
Kidneys
Kidney cortex samples
Omics data
RNA sequencing
Microarray
Proteomics
RNA sequencing
b
Over 20 artificial intelligence and bioinformatic models are used to rank target-disease association
Omics AI scores
Network Neighbors
Mutated Sub-modules
Disease Sub-modules
Causal Inference
Overexpression
Knockouts
Mutations
Pathways
InteractomeCommunity
Relevance
Expression
Matrix Factorization
Heterogenous Graph Walk
Text-based AI scores
Attention Spike
Evidence
Attention Score
Trend
KOL scores
Credible Attention Index
Mean Hirsch
Impact Factor
Financial scores
Funding per Publication
Grant Funding
Grant Size
AI
Druggability assessment
Clinical development
Tissue Specificity
Target identification
c
RANK
Network Neighbors
Mutated Sub-modules
Disease Sub-modules
Causal Inference
Overexpression
Knockouts
Mutations
Pathways
Interactome Community
Relevance
Expression
Heterogenous Graph Walk
Matrix Factorization
Attention Spike
Evidence
Attention Score
Trend
Funding per Publication
Grant Funding
Grant Size
Credible Attention Index
Mean Hirsch
Impact Factor
Tissue specificity
Specificity in the Selected Tissue
Small Molecule
Antibodies
Safety
Novelty
Structure
Development Level
Target Family
MTOR Tclin Protein Kinase Other
PLK1 Tchem Protein Kinase Other
CXCR4 Tclin GPCR
JAK2 Tclin Tyrosine Kinase
ITGB2 Tclin Receptor Other
REN Tclin Peptidase
ACLY Tclin Acyltransferase
LIG1 Tchem Ligase
CDK6 Tclin CMGC Kinase
PIK3CD Tclin Non-Protein Kinase
RANK
OMICS SCORES
TEXT-BASED
FINANCIAL
KOL
TISSUE

Supplementary Figure 6. Artificial intelligence (AI)-powered pipeline to identify drug targets in animal- and cell-based models of cystinosis. An end-to-end AI-driven engine uses machine learning (ML) tools and statistical validation to rank disease-target associations and prioritize actionable (drug) targets in the tubular cells lacking CTNS and accumulating cystine. The AI-powered target discovery platform (henceforth PandaOmics) extracts disease knowledge from a variety of sources that include (**a**) multi-omics landscapes, here derived from transcriptomics-based signatures in the kidney cortex samples obtained from both *Ctns* mouse and rat, or from proteomics-based profiling of mPTCs derived from *Ctns* mouse kidneys, and (**b**) text-curated data (patents, grants, publications, clinical trials, and key opinion leaders). The combination of described scores results in a ranked list of targets proposed for a given disease and can be filtered out through the assessment of druggability properties that include safety, novelty, accessibility by approved and/or investigational small molecules or biologics, and tissue specificity. (**c**) Heat map showing the top 10 most promising (drug) targets reversing dysregulated homeostasis in CTNS-deficient/cystinosis-affected tubular cells, with mTOR being identified as one of the top hits among the list of predicted targets. Images in **a** were drawn using pictures from Servier Medical Art, which is licensed under a Creative Commons Attribution 3.0 Unported License (https://creativecommons.org/licenses/by/3.0/).

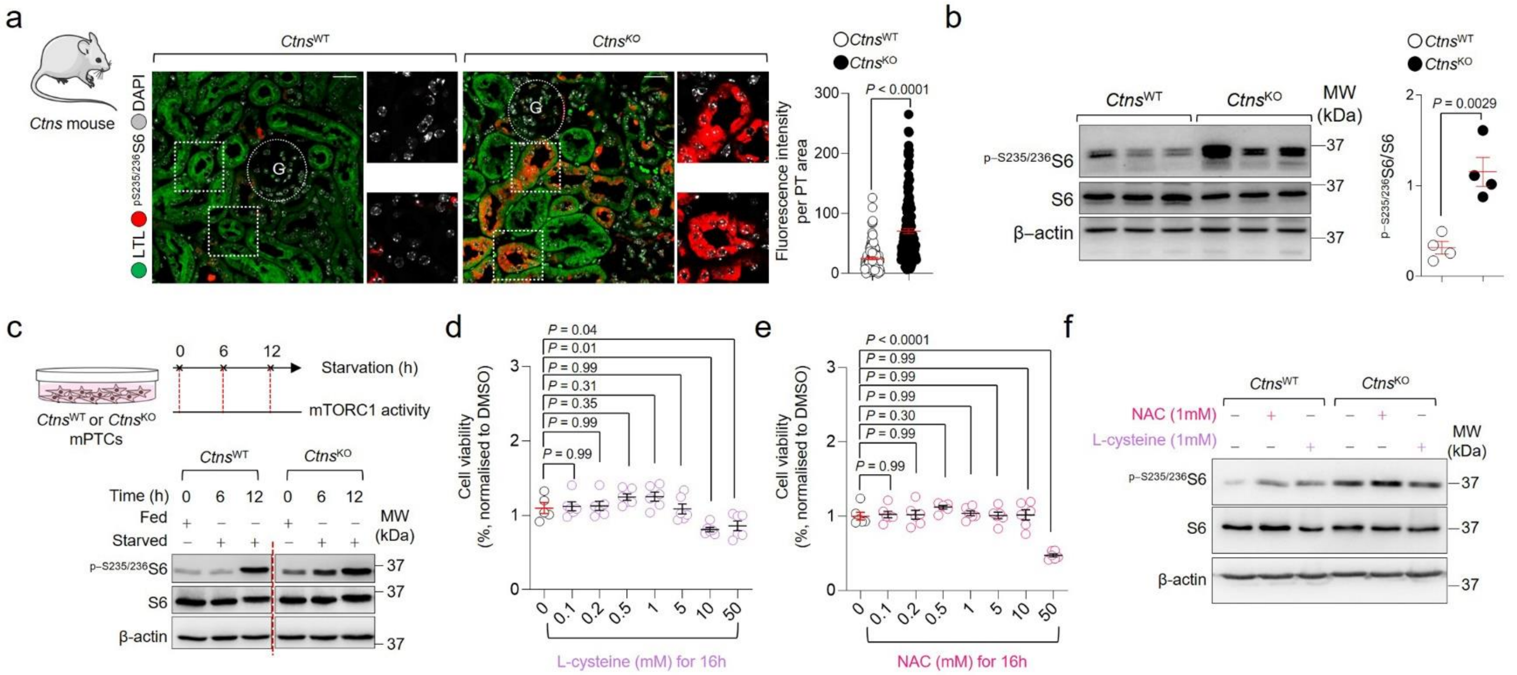

Supplementary Figure 7. mTORC1 signaling landscape in the PT segments of *Ctns* mouse kidneys and derived tubular cells. (**a**) Representative confocal images and quantification of mean fluorescence intensity of phosphorylated $^{pS235/236}$S6 in the LTL^{+} (Lotus Tetragonolobus Lectin, green) PT segments of 24-week-old *Ctns* mouse kidneys; n=131 *Ctns*^{WT} and n=170 *Ctns*^{KO} PT segments pooled from 3 biologically independent animals. (**b**) Immunoblot and quantification of the indicated proteins in the kidneys of 24-week-old *Ctns* mouse kidneys; n=4 biologically independent animals in each experimental group. (**c**) *Ctns* mPTCs were fed or starved for the indicated times and the whole cell lysates were immunoblotted for the indicated proteins and phosphoproteins; n=2 biologically independent experiments. (**d** and **e**) *Ctns*^{WT} mPTCs were treated with (**d**) L-cysteine or (**e**) (N-acetyl cysteine, NAC) at the indicated concentrations. After 16h treatment, the cell viability was assessed by MTT assay. The cell viability was normalized using the mean of the corresponding control; n=6 wells pooled from 2 biologically independent experiments. (**f**) *Ctns* mPTCs were treated with non-toxic concentrations of L-cysteine (1mM) and NAC (1mM) for 16h. Whole cell lysates were immunoblotted for the indicates proteins and phosphoproteins; n=2 biologically independent experiments. Plots represent mean ± SEM. Statistical calculated by unpaired two-tailed Student's *t* test in **a** and **b** and by one-way ANOVA followed by Dunnett's multiple comparisons test in **d** and **e**. Scale bars are 50μm. Source data are provided as a Source Data file. Images in **a** and **c** were drawn using pictures from Servier Medical Art, which is licensed under a Creative Commons Attribution 3.0 Unported License (https://creativecommons.org/licenses/by/3.0/).

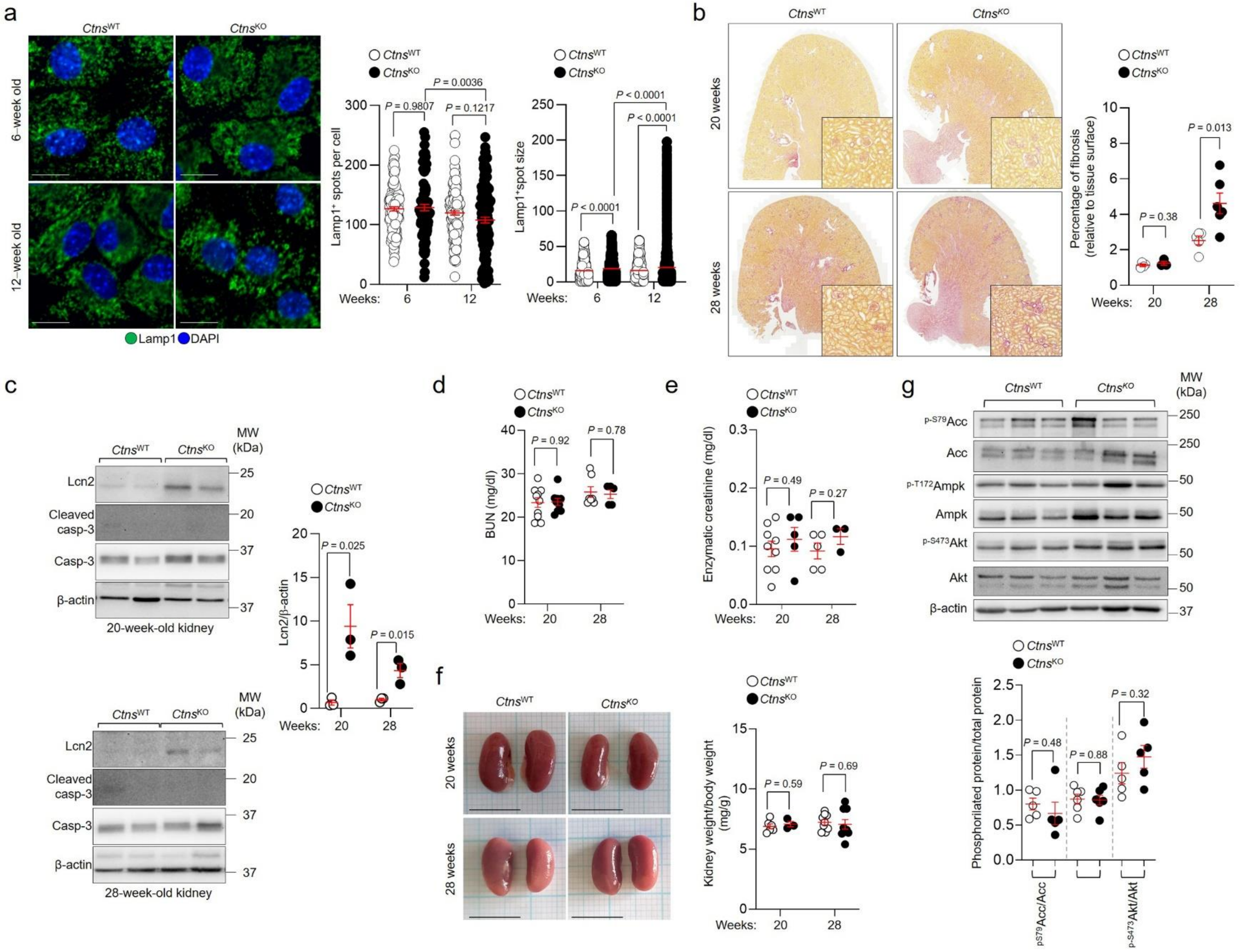
a
CtnsWT
CtnsKO
6-week old
12-week old
Lamp1 DAPI
Lamp1+ spots per cell
P = 0.9807
P = 0.0036
P = 0.1217
Weeks: 6 12
Lamp1+spot size
P < 0.0001
Weeks: 6 12
b
20 weeks
28 weeks
Percentage of fibrosis (relative to tissue surface)
P = 0.38
P = 0.013
Weeks: 20 28
c
Lcn2
Cleaved casp-3
Casp-3
β-actin
MW (kDa)
20-week-old kidney
28-week-old kidney
Lcn2/β-actin
P = 0.025
P = 0.015
d
BUN (mg/dl)
P = 0.92
P = 0.78
e
Enzymatic creatinine (mg/dl)
P = 0.49
P = 0.27
f
Kidney weight/body weight (mg/g)
P = 0.59
P = 0.69
g
p-S79Acc
Acc
p-T172Ampk
Ampk
p-S473Akt
Akt
Phosphoriliated protein/total protein
P = 0.48
P = 0.88
P = 0.32
pS79Acc/Acc
p-S473Akt/Akt

Supplementary Figure 8. Phenotypic changes in the mouse kidneys lacking CTNS and accumulating cystine. (**a**) mPTCs derived from 6- and 12-week-old *Ctns* mouse kidneys were immunostained for Lamp1 and analysed by confocal microscopy. Representative images and quantification of the number of Lamp1$^+$ structures and size. Number of Lamp1$^+$ spots: n=123 *Ctns*^{WT} and n=96 *Ctns*^{KO} cells, and n=139 *Ctns*^{WT} and n=141 *Ctns*^{KO} cells from 6-week- and12-week-old mouse kidneys, respectively; Size of Lamp1$^+$ spots: n=14647 *Ctns*^{WT} and n= 13337 *Ctns*^{KO} lysosomes, and n=14356 *Ctns*^{WT} and n=17830 *Ctns*^{KO} lysosomes analysed in the mPTCs from 6-week- and 12-week-old mouse kidneys, respectively. The cells used in these analyses were pooled from 2 biologically independent animals. (**b**) Representative micrographs of Picrosirius Red staining and quantification of fibrosis in the kidneys of 20- and 28-week-old *Ctns* mice; n=4 *Ctns*^{WT} and n=3 *Ctns*^{KO} animals, and =5 *Ctns*^{WT} and n=6 *Ctns*^{KO} animals at 24 and 28 weeks of age, respectively. Black insets contain images at high magnification. (**c**) Immunoblotting and quantification of the indicated proteins; n=3 biologically independent animals in each experimental group. (**d** and **e**) Quantification of the levels of (**d**) blood urea nitrogen (BUN) and (**e**) enzymatic creatinine in the plasma samples of *Ctns* mice at the indicated times. For BUN: n=10 *Ctns*^{WT} and n=9 *Ctns*^{KO} animals, and n=7 *Ctns*^{WT} and n=5 *Ctns*^{KO} animals at 20 and 28 weeks of age, respectively. For enzymatic creatinine: n=9 *Ctns*^{WT} and n=5 *Ctns*^{KO} animals, and n=5 *Ctns*^{WT} and n=3 *Ctns*^{KO} animals at 20 and 28 weeks of age, respectively. (**f**) Representative images of *Ctns* mouse kidneys and ratio of kidney weight to body weight at the indicated times; n=6 *Ctns*^{WT} and n=3 *Ctns*^{KO}, and n=9 *Ctns*^{WT} and *Ctns*^{KO} animals at 20 and 28 weeks of age, respectively. Each dot of the graph represents the average of the two kidneys derived from one mouse. (**g**) Immunoblotting and quantification of the indicated proteins and phosphoproteins in the kidneys of 24-week-old *Ctns* mice; for Acc and Akt: n=5 *Ctns*^{WT} and *Ctns*^{KO}, and for Ampk: n=6 *Ctns*^{WT} and Ctns^{KO} biologically independent animals at 20 and 28 weeks of age, respectively. Plotted data represent mean ±SEM. Statistical calculated by and by one-way ANOVA followed by Sidak's multiple comparisons test in **a** and unpaired two-tailed Student's *t* test in **b**-**g**. Scale bars are 10μm. Source data are provided as a Source Data file.

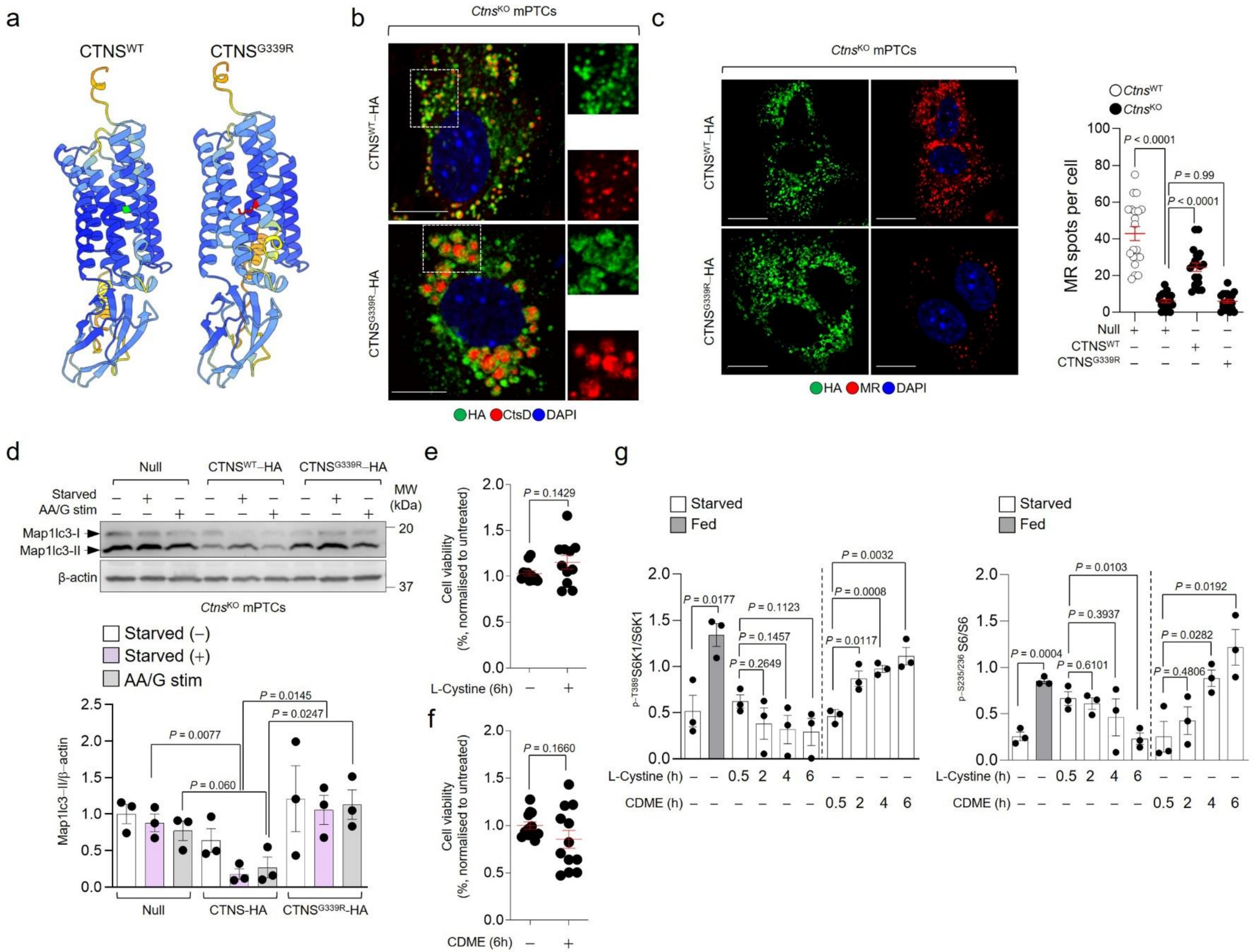
a
CTNSWT
CTNSG339R
b
CtnsKO mPTCs
CTNSWT–HA
CTNSG339R–HA
HA CtsD DAPI
c
CtnsKO mPTCs
CTNSWT–HA
CTNSG339R–HA
HA MR DAPI
CtnsWT
CtnsKO
MR spots per cell
P < 0.0001
P = 0.99
P < 0.0001
Null
CTNSWT
CTNSG339R
d
Null
CTNSWT–HA
CTNSG339R–HA
Starved
AA/G stim
MW (kDa)
Map1lc3-I
Map1lc3-II
β-actin
CtnsKO mPTCs
Starved (–)
Starved (+)
AA/G stim
Map1lc3–II/β–actin
P = 0.0145
P = 0.0247
P = 0.0077
P = 0.060
Null
CTNS-HA
CTNSG339R-HA
e
Cell viability (%, normalised to untreated)
P = 0.1429
L-Cystine (6h)
f
P = 0.1660
CDME (6h)
g
Starved
Fed
p-T389S6K1/S6K1
P = 0.0177
P = 0.1123
P = 0.1457
P = 0.2649
P = 0.0032
P = 0.0008
P = 0.0117
L-Cystine (h)
CDME (h)
p-S235/236 S6/S6
P = 0.0103
P = 0.3937
P = 0.0004
P = 0.6101
P = 0.0192
P = 0.0282
P = 0.4806

Supplementary Figure 9. Lysosomal cystine levels regulate mTORC1 in the tubular cells lining the kidney PT segments. (**a**) Protein-structure prediction of $CTNS^{WT}$ and $CTNS^{G339R}$ by using the neural network AlphaFold. (**b**-**c**) mPTCs from 24-week-old *Ctns*^{KO} mouse kidneys were transduced with adenoviral particles expressing a Null or HA-tagged wild-type CTNS ($CTNS^{WT}$-HA) or HA-tagged mutant CTNS ($CTNS^{G339R}$-HA) for 2 days. (**b**) Cells were immunostained for the epitope HA (green) and cathepsin D (CtsD, red), and analyzed by confocal microscopy. Dotted white squares contain images at high magnification. The experiments in **b** were performed two times independently and all attempts were successful with similar results.(**c**) Cells were pulsed with MagicRed Cathepsin B substrate (MR, 1μM at 37°C for 1h) and immunostained for HA and analyzed by confocal microscopy. Representative images and quantification of MR-positive structures in *Ctns* mPTCs; n=20 transduced cells expressing HA from 2 biologically independent samples were analysed in each experimental group. (**d**) *Ctns* mPTCs were cultured under fed or starved conditions or restimulated by adding back amino acids and glucose (AA/G stim). Immunoblotting and quantification of the indicated proteins in the mouse tubular cells lacking CTNS; n=3 biologically independent experiments in each experimental group. (**e**-**g**) Starved *Ctns*^{WT} mPTCs were stimulated with cystine dimethyl ester (CDME, 0.1 mM) or with unmodified L-cystine (0.1 mM) for the indicated times. (**e**-**f**) MTT assay monitoring cell viability in (un)treated *Ctns* mPTCs. Cell viability was normalized using the mean of the corresponding control; n= 11 wells (for L-cystine) and n=12 wells (for CDME) pooled from 2 biologically independent experiments. (**g**) Quantification of immunoblotting shown in Fig. 5h. Plotted data represent mean ± SEM. Statistical calculated by unpaired two-tailed Student's *t* test in **d**, **e**, **f**, and **g**,and by one-way ANOVA followed by Tukey's multiple comparisons test in **c**. Nuclei counterstained with DAPI (blue). Scale bars are 10µm. Source data are provided as a Source Data file.

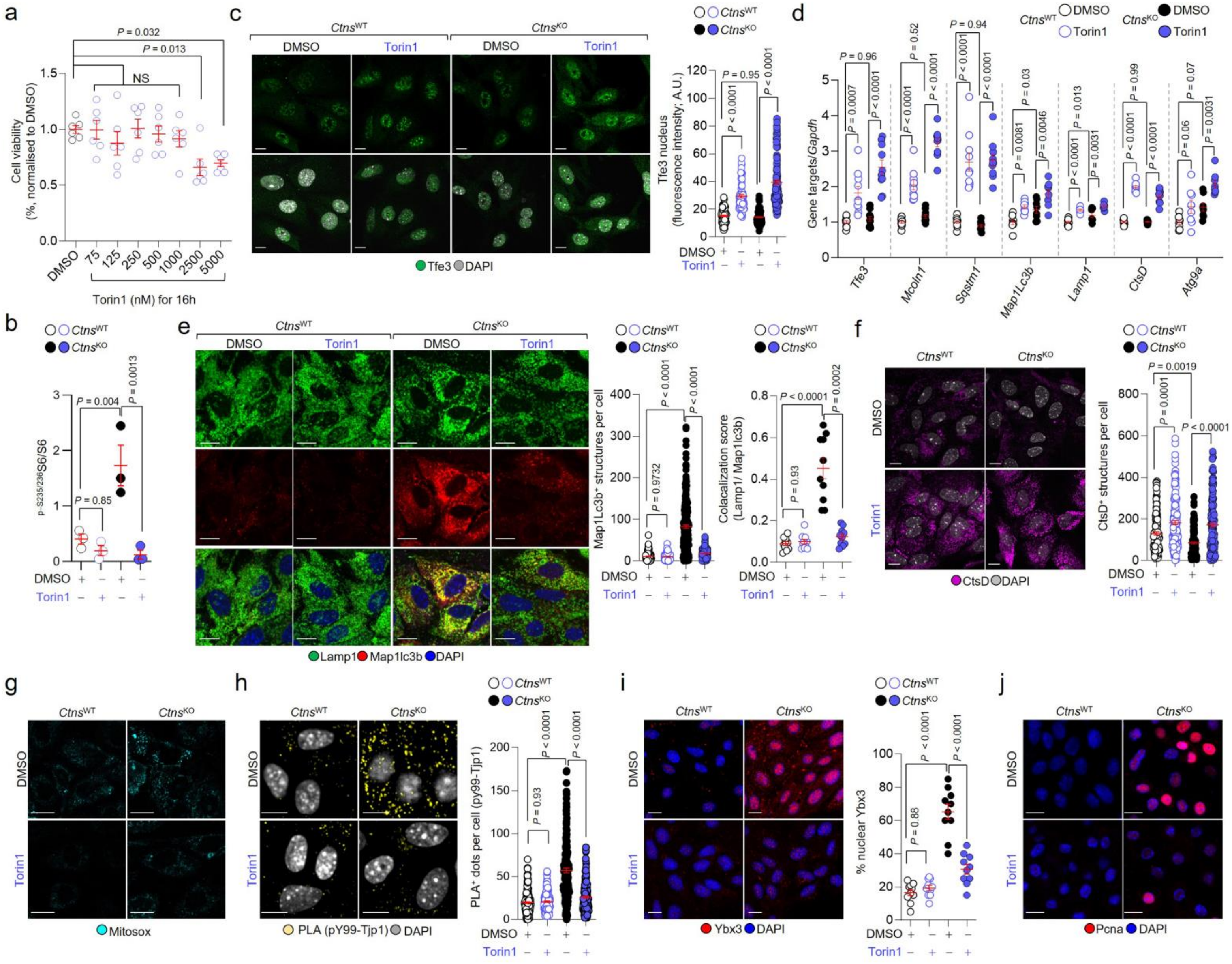

a
P = 0.032
P = 0.013
NS
Cell viability
(%, normalised to DMSO)
DMSO
75
125
250
500
1000
2500
5000
Torin1 (nM) for 16h
b
CtnsWT
CtnsKO
P = 0.004
P = 0.0013
P = 0.85
p-S235/236S6/S6
DMSO
Torin1
c
CtnsWT
CtnsKO
DMSO
Torin1
Tfe3 DAPI
P = 0.95
P < 0.0001
Tfe3 nucleus
(fluorescence intensity; A.U.)
d
CtnsWT DMSO
Torin1
CtnsKO DMSO
Torin1
Gene targets/Gapdh
Tfe3
Mcoln1
Sqstm1
Map1Lc3b
Lamp1
CtsD
Atg9a
P = 0.96
P = 0.0007
P < 0.0001
P = 0.52
P = 0.94
P = 0.03
P = 0.0081
P = 0.0046
P = 0.013
P = 0.0031
P = 0.99
P = 0.07
P = 0.06
e
CtnsWT
CtnsKO
DMSO
Torin1
Lamp1 Map1lc3b DAPI
Map1Lc3b+ structures per cell
P = 0.9732
P < 0.0001
Colacalization score
(Lamp1/ Map1lc3b)
P = 0.93
P = 0.0002
f
CtnsWT
CtnsKO
DMSO
Torin1
CtsD DAPI
CtsD+ structures per cell
P = 0.0019
P = 0.0001
P < 0.0001
g
CtnsWT
CtnsKO
DMSO
Torin1
Mitosox
h
CtnsWT
CtnsKO
DMSO
Torin1
PLA (pY99-Tjp1) DAPI
PLA+ dots per cell (py99-Tjp1)
P = 0.93
P < 0.0001
i
CtnsWT
CtnsKO
DMSO
Torin1
Ybx3 DAPI
% nuclear Ybx3
P = 0.88
P < 0.0001
j
CtnsWT
CtnsKO
DMSO
Torin1
Pcna DAPI

Supplementary Figure 10. The pharmacological inhibition of mTORC1 signaling rescues lysosome catabolism and abrogates abnormal programs for growth while restoring differentiation in CTNS-deficient mPTCs. (**a**) MTT assay measuring cell viability in Torin1-treated and untreated *Ctns*^{WT} mPTCs. The cell viability normalized using the mean of the corresponding DMSO control; n=6 wells pooled from 2 biologically independent experiments. (**b**-**h**) mPTCs were treated with DMSO or Torin1 (250nM, for 16h). (**b**) Quantification of the immunoblotting shown in Fig. 6a. (**c**) Representative confocal images and quantification of mean fluorescence intensity of nuclear TFE3 signal; n=69 (*Ctns*^{WT} + DMSO), n=82 (*Ctns*^{WT} + Torin1), n=111 (*Ctns*^{KO} + DMSO) and n=132 (*Ctns*^{KO} + Torin1) cells pooled from two biologically independent animals. (**d**) mRNA levels of the indicated genes (*Mcoln1*, *Sqstm1* and *Map1Lc3B*: n=10 wells; *Lamp1*, *CstD* and *Atg9a*: n=8 wells pooled from 3 biologically independent experiments. (**e**) Representative confocal micrographs and quantification of the number of Map1Lcb3^{+} puncta and Map1lc3-Lamp1 colocalization in *Ctns* mPTCs. For Map1Lc3b^{+} structures: n=283 (*Ctns*^{WT} + DMSO), n=254 (*Ctns*^{WT} + Torin1), n=229 (*Ctns*^{KO} + DMSO), and n=284 (*Ctns*^{KO} + Torin1) cells pooled from 2 biologically independent experiments. For colocalization score: n=10 randomly selected and non-overlapping fields of views in each experimental group. (**f**) Representative confocal images and quantification of the number of CtsD-positive structures in *Ctns* mPTCs; n=137 (*Ctns*^{WT} + DMSO), n=167 (*Ctns*^{WT} + Torin1), n=138 (*Ctns*^{KO} + DMSO), and n=181 (*Ctns*^{KO} + Torin1) cells pooled from 2 biologically independent animals. (**g**) *Ctns* mPTCs were loaded with MitoSOX (mitochondrial ROS probe; 2.5μM for 10 min at 37 °C for 1h) and analysed by confocal microscopy. Representative confocal images of the quantification shown in Fig. 6e. (**h**) Confocal analysis of endogenous phosphorylation of Tjp1 by proximity ligation assay (PLA) and quantification of PLA^{+} puncta in *Ctns* mPTCs; n=181 (*Ctns*^{WT} + DMSO), n=154 (*Ctns*^{WT} + Torin1), n=166 (*Ctns*^{KO} + DMSO), and n=235 (*Ctns*^{KO} + Torin1) cells pooled from 2 biologically independent experiments. (**i**) Representative confocal images and quantification of the number Ybx3-positive nuclei; n=10 randomly selected and non-overlapping fields of views in each experimental group. (**j**) Representative confocal images of the quantification shown in Fig. 6g. All values were pooled from 2 biologically independent experiments in **e** (colocalization score) and **i.** Nuclei counterstained with DAPI (grey or blue). Plotted data represent mean ± SEM. Statistics calculated by one-way ANOVA followed Dunnett's multiple comparisons test in **a**, by one-way ANOVA followed Sidak's multiple comparisons test in **b**, **c**, and **d** and **f**, **g**, **h**, and **i** by one-way ANOVA followed Tukey's multiple comparisons test in **e.** Scale bars are 10μm. NS, non-significant. Source data are provided as a Source Data file.

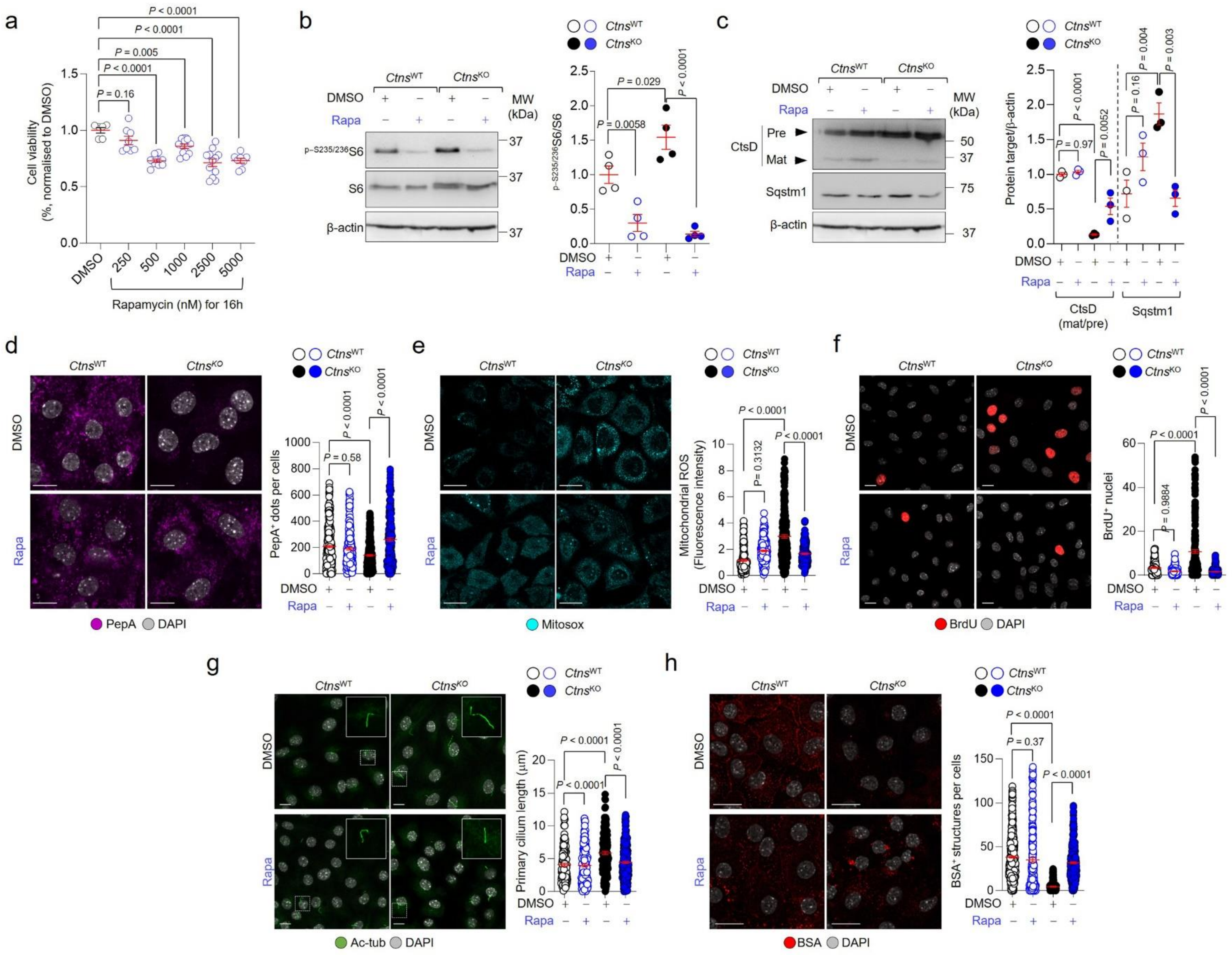
a
b
c
d
e
f
g
h
Cell viability (%, normalised to DMSO)
Rapamycin (nM) for 16h
DMSO
250
500
1000
2500
5000
P = 0.16
P < 0.0001
P = 0.005
Ctns^WT
Ctns^KO
DMSO
Rapa
MW (kDa)
p-S235/236S6
S6
β-actin
p-S235/236S6/S6
P = 0.0058
P = 0.029
CtsD
Pre
Mat
Sqstm1
Protein target/β-actin
P = 0.97
P = 0.0052
P = 0.004
P = 0.003
CtsD (mat/pre)
PepA DAPI
PepA+ dots per cells
P = 0.58
Mitosox
Mitochondrial ROS (Fluorescence intensity)
P = 0.3132
BrdU DAPI
BrdU+ nuclei
P = 0.9884
Ac-tub DAPI
Primary cilium length (µm)
BSA DAPI
BSA+ structures per cells
P = 0.37

Supplementary Figure 11. Rapamycin restores the catabolic activities of the lysosome and improves PT cell differentiation downstream of cystine storage. (**a**) MTT assay measuring cell viability in Rapamycin-treated and untreated mPTCs. The cell viability normalized using the mean of the corresponding DMSO control; n= 6 (*Ctns*^{WT} + DMSO), n=8 (*Ctns*^{WT} + 250 nM Rapa), n=12 (*Ctns*^{WT} + 500 nM Rapa), n= 12 (*Ctns*^{WT} + 1000 nM Rapa) and n=10 (*Ctns*^{WT} + 2500 nM Rapa), and n=8 (*Ctns*^{WT} + 2500 nM Rapa) wells pooled from 2 biologically independent experiments. (**b-h**) *Ctns* mPTCs were treated with low, non-toxic concentration of Rapamycin (250 nM for 16h). **(b-c**) Immunoblotting and quantification of the indicated proteins and phosphoproteins; For S6: n=4 biologically independent samples and for CtsD and Sqstm1: n=3 biologically independent samples in each experimental group, respectively. (**d**) The cells were pulsed for 1h with Pepstatin A (PepA;1μM at 37°C, pink) and analysed by confocal microscopy. Representative confocal images and quantification of the number of PepstatinA^{+} structures in *Ctns* mPTCs; n=315 (*Ctns*^{WT} + DMSO), n=221 (*Ctns*^{WT} + Rapa), n=314 (*Ctns*^{KO} + DMSO), and n=225 (*Ctns*^{KO} + Rapa) cells pooled from 2 biologically independent animals. (**e**) Confocal microscopy and quantification of MitoSOX fluorescence intensity in *Ctns* mPTCs; n=126 (*Ctns*^{WT} + DMSO), n=167 (*Ctns*^{WT} + Rapa), n=226 (*Ctns*^{KO} + DMSO), and n=209 (*Ctns*^{KO} + Rapa) cells pooled from 2 biologically independent animals. (**f**) Confocal microscopy and quantification of BrdU^{+} cells (expressed as percentage of total cells); n=112 (*Ctns*^{WT} + DMSO), n=145 (*Ctns*^{WT} + Rapa), n=207 (*Ctns*^{KO} + DMSO), and n=206 (*Ctns*^{KO} + Rapa) pooled from 2 biologically independent animals. (**g**) Maximum intensity projection of image stacks and quantification of the primary cilium length (Ac-tubulin, green) in *Ctns* mPTCs; n=135 (*Ctns*^{WT} + DMSO), n=154 (*Ctns*^{WT} + Rapa), n=153 (*Ctns*^{KO} + DMSO), and n=303 (*Ctns*^{KO} + Rapa) cells pooled from 2 biologically independent animals. (**h**) Cells were pulsed with Alexa633-labelled BSA for 15 min and analyzed by confocal microscopy. Representative confocal images and quantification of the number of BSA-positive structures in *Ctns* mPTCs; n=548 (*Ctns*^{WT} + DMSO), n=229 (*Ctns*^{WT} + Rapa), n=130 (*Ctns*^{KO} + DMSO), and n=352 (*Ctns*^{KO} + Rapa) cells pooled from 2 biologically independent animals. Plots represent mean ± SEM. All values were pooled from 3 biologically independent experiments in **d**, **e**, **f**, **g** and **h**. Nuclei counterstained with DAPI (grey). Statistics calculated by one-way ANOVA followed Dunnett's multiple comparisons test in **a, b** and by one-way ANOVA followed by Sidak's or Tukey's comparisons multiple comparison test in **c**, **d**, **e**, **f**, **g,** and **h**. Scale bars are 10μm. Source data are provided as a Source Data file.

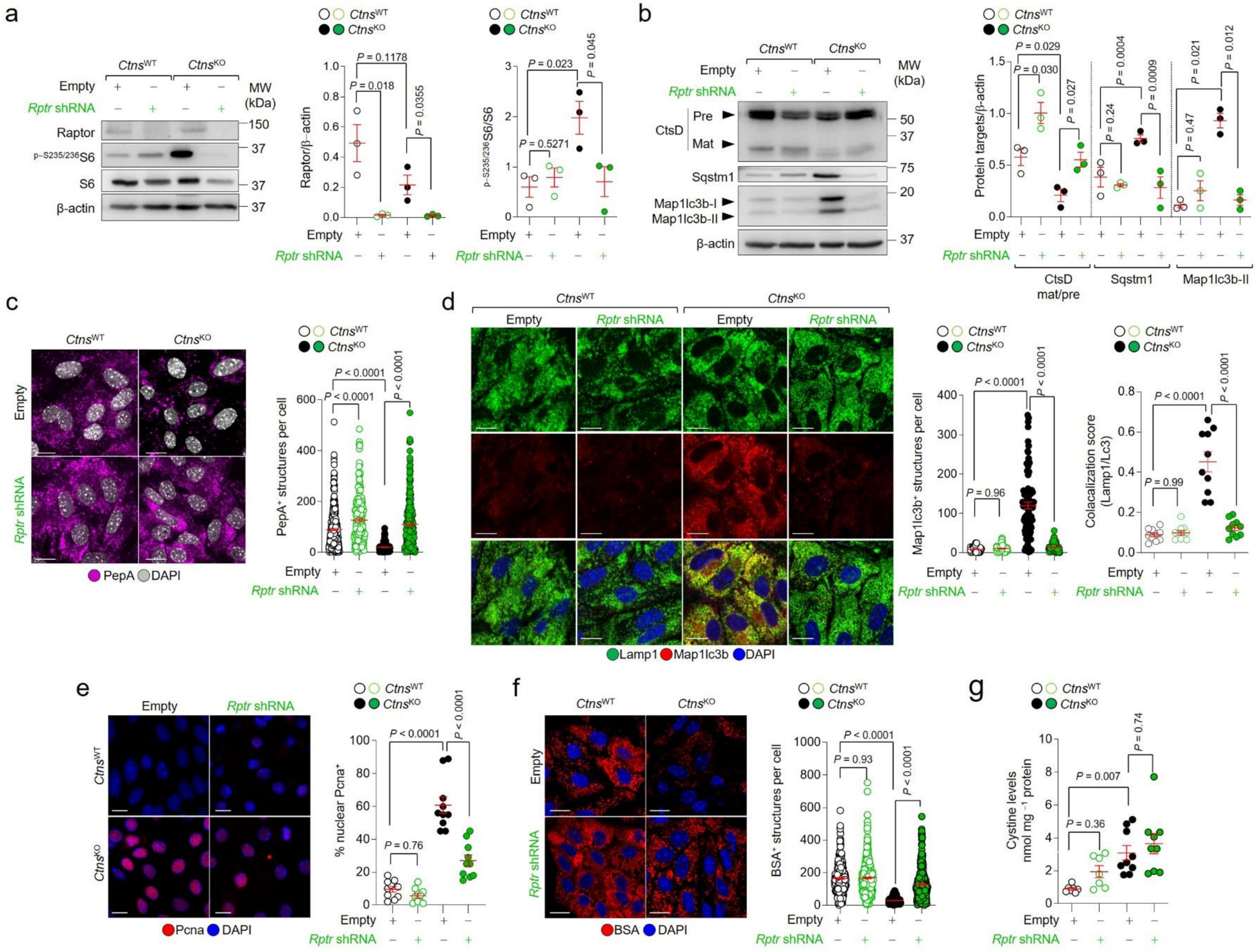

Supplementary Figure 12. *Raptor* knockdown rescues lysosomal storage-related phenotypes and epithelial function in CTNS-deficient mPTCs. (**a**-**g**) Cells were transduced with adenovirus particles bearing Empty or *Raptor*-shRNA (*Rptr* shRNA) for 5 days. (**a**-**b**) Immunoblotting and quantification of the indicated proteins and phosphoproteins; n=3 biologically independent samples. (**c**) The transduced *Ctns* mPTCs were pulsed with Pepstatin A (PepA;1μM at 37°C for 1h, pink) and analysed by confocal microscopy. Representative confocal images and quantification of the number of PepstatinA-positive structures in *Ctns* mPTCs; n=361 ($Ctns^{WT}$ + Empty), n=227 ($Ctns^{WT}$ + *Rptr* shRNA), n=258 ($Ctns^{KO}$ + Empty), and n=256 ($Ctns^{KO}$ + *Rptr* shRNA) cells pooled from 2 biologically independent animals. (**d**) Representative confocal images and quantification of the number of Map1Lc3b-positive structures and Map1Lc3b-Lamp1 colocalization. For $Map1Lc3b^{+}$ structures: n=90 cells pooled from 3 biologically independent animals. For colocalization score: n=10 randomly selected and non-overlapping fields of views in each experimental group. (**e**) Representative confocal images and quantification of the number of $Pcna^{+}$ nuclei; n=10 randomly selected and non-overlapping fields of views in each experimental group. (**f**) Cells were pulsed with Alexa633-labelled BSA for 15 min and analyzed by confocal microscopy. Representative confocal images and quantification of the number of BSA-positive structures per cell; n=324 ($Ctns^{WT}$ + Empty), n=437 ($Ctns^{WT}$ + *Rptr* shRNA), n=199 ($Ctns^{KO}$ + Empty), and n=305 ($Ctns^{KO}$ + *Rptr* shRNA) cells pooled from 3 biologically independent animals. (**g**) Cystine levels by HPLC; n=7 ($Ctns^{WT}$ + Empty) and ($Ctns^{WT}$ + *Rptr* shRNA), n=9 ($Ctns^{KO}$ + Empty) and ($Ctns^{KO}$ + *Rptr* shRNA) wells pooled from 3 biologically independent animals. Plots represent mean ± SEM. All values were pooled from three biologically independent experiments in **c**, **d**, **e**, **f,** and **g**. Nuclei counterstained with DAPI (blue or grey). Statistics calculate by unpaired two-tailed Student's *t* test in **a** and **b,** and by one-way ANOVA followed by Sidak's or Tukey's comparisons in **c**, **d**, **e**, **f,** and **g**. Scale bars are 10μm. Source data are provided as a Source Data file.

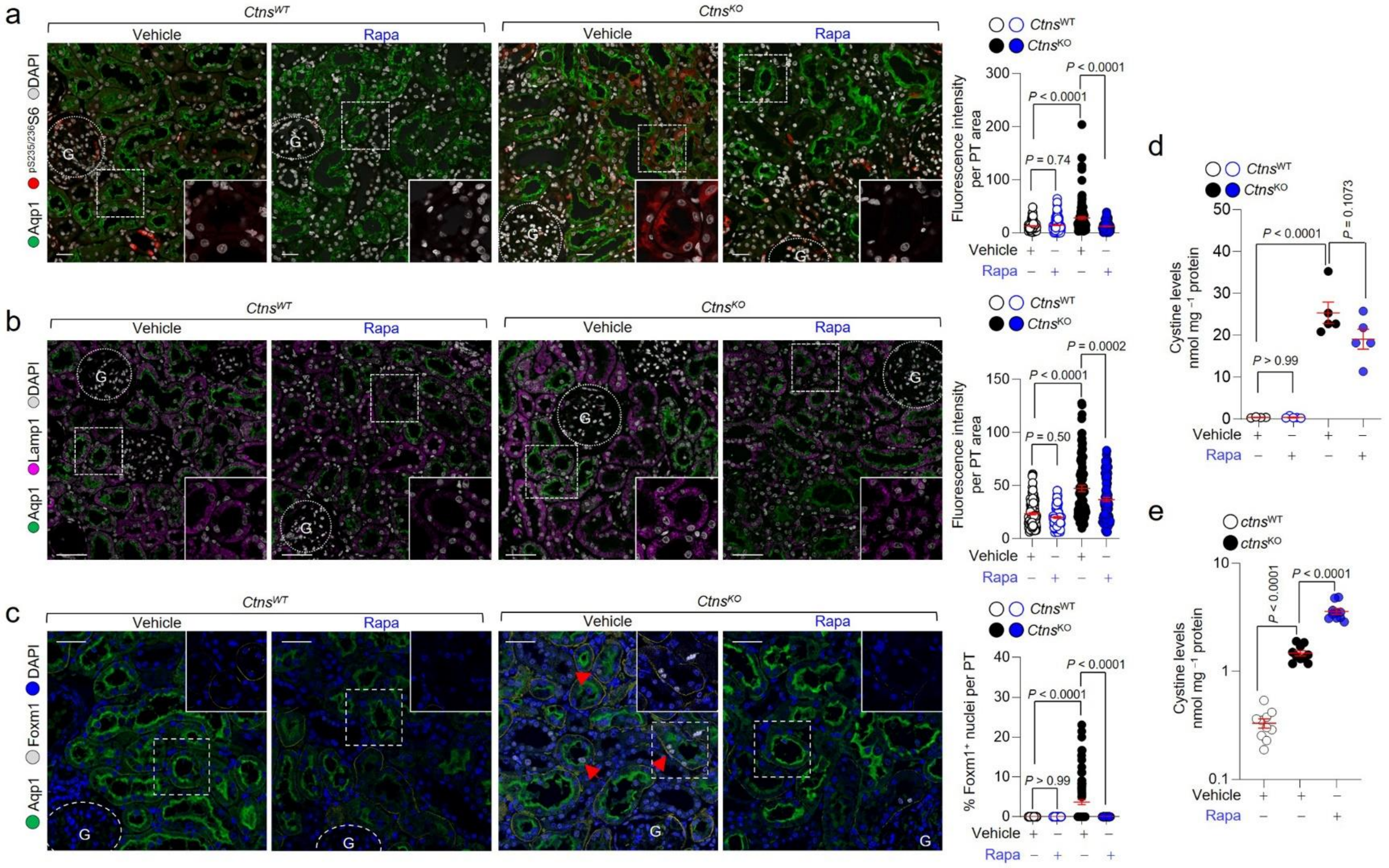

Supplementary Figure 13. Effects of Rapamycin in the PT segments of *Ctns*-deficient rat kidneys and in *ctns*-deficient zebrafish. (**a**-**d**) *Ctns* rats at 12 weeks of age were subcutaneously implanted with a pellet providing a long-term release of Rapamycin (Rapa, 1.5 mg/kg/day). After 2 weeks of treatment, the kidneys were harvested, immunostained for the indicated markers and analysed by confocal microscopy. (**a**-**c**) Representative confocal images and quantification of fluorescence intensity of (**a**) $^{pS235/236}$S6 (red) or (**b**) Lamp1 (pink) or (**c**) quantification of the number of Foxm1^{+} nuclei (gray) in Aqp1-positive PT segments (green) of *Ctns* rat kidneys. For S6: n=150 (*Ctns*^{WT} + Vehicle), n=199 (*Ctns*^{WT} + Rapa), n= 121 (*Ctns*^{KO} + Vehicle), and n=200 (*Ctns*^{KO} + Rapa)

PT segments; for Lamp1: n=154 (*Ctns*^{WT} + Vehicle), n=94 (*Ctns*^{WT} + Rapa), n= 94 (*Ctns*^{KO} + Vehicle), and n=128 (*Ctns*^{KO} + Rapa) PT segments; For Foxm1: n=119 (*Ctns*^{WT} + Vehicle); n=86 (*Ctns*^{WT} + Rapa), n= 104 (*Ctns*^{KO} + Vehicle), and n=93 (*Ctns*^{KO} + Rapa) PT segments. All the values were pooled from 3 biologically independent animals. (**d**) Quantification of cystine levels by HPLC in the *Ctns* rat kidneys; n=5 biologically independent animals in each experimental group. (**e**) 5dpf-*ctns* zebrafish larvae were treated with Vehicle or Rapamycin (200nM) for 9 days. Quantification of cystine levels by HPLC in the zebrafish larvae, n=10 biologically independent samples in each experimental group, with each point representing a pool of seven zebrafish. Statistics calculate by one-way ANOVA followed by Tukey's multiple comparison test in **a, b**, cand **d**, and by one-way ANOVA followed by Holm-Sidak's multiple comparisons test in **e.** Scale bars are 50μm. Source data are provided as a Source Data file.

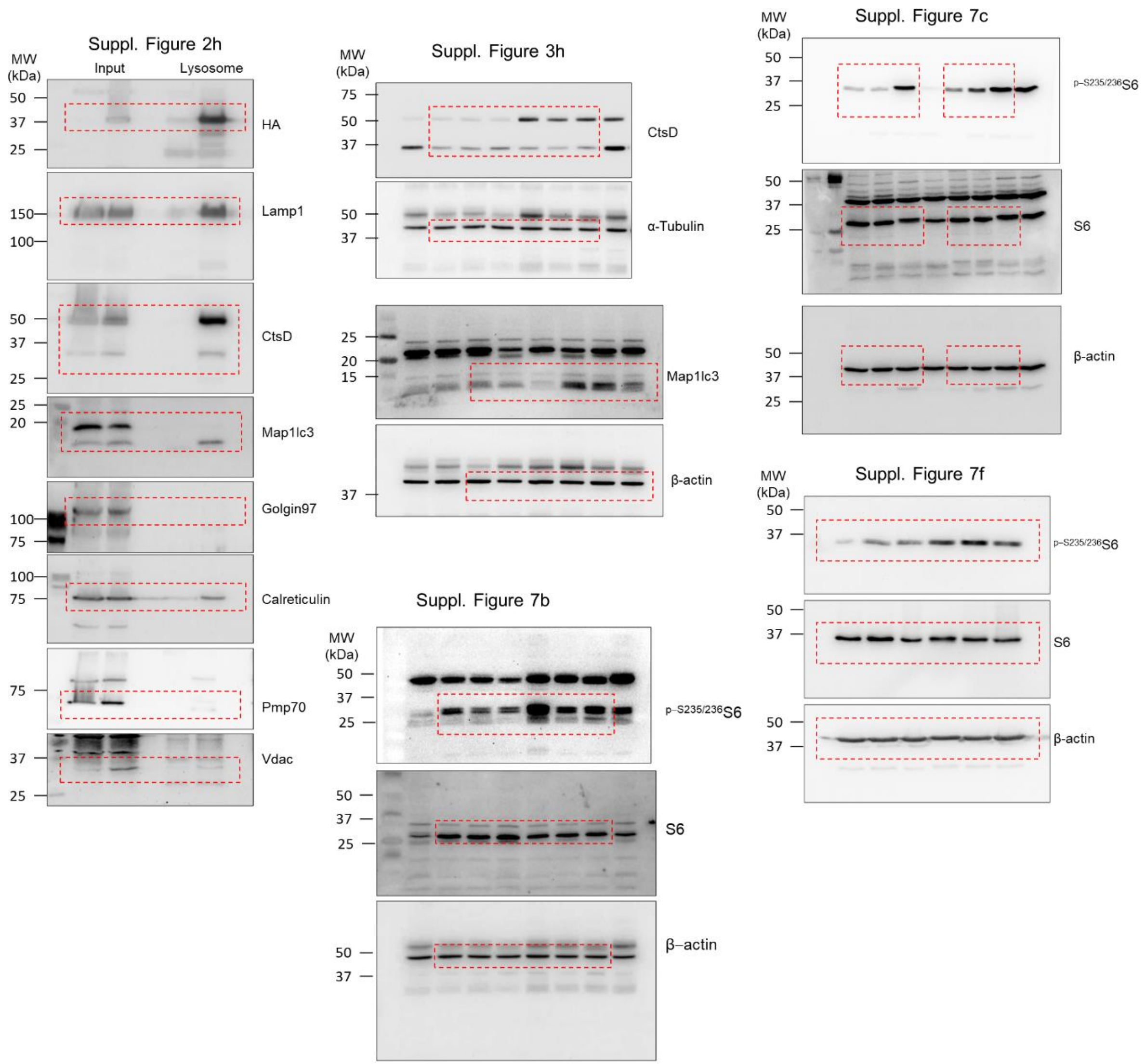

Supplementary Fig. 14. Uncropped and unprocessed versions of western blots presented in the supplementary figures

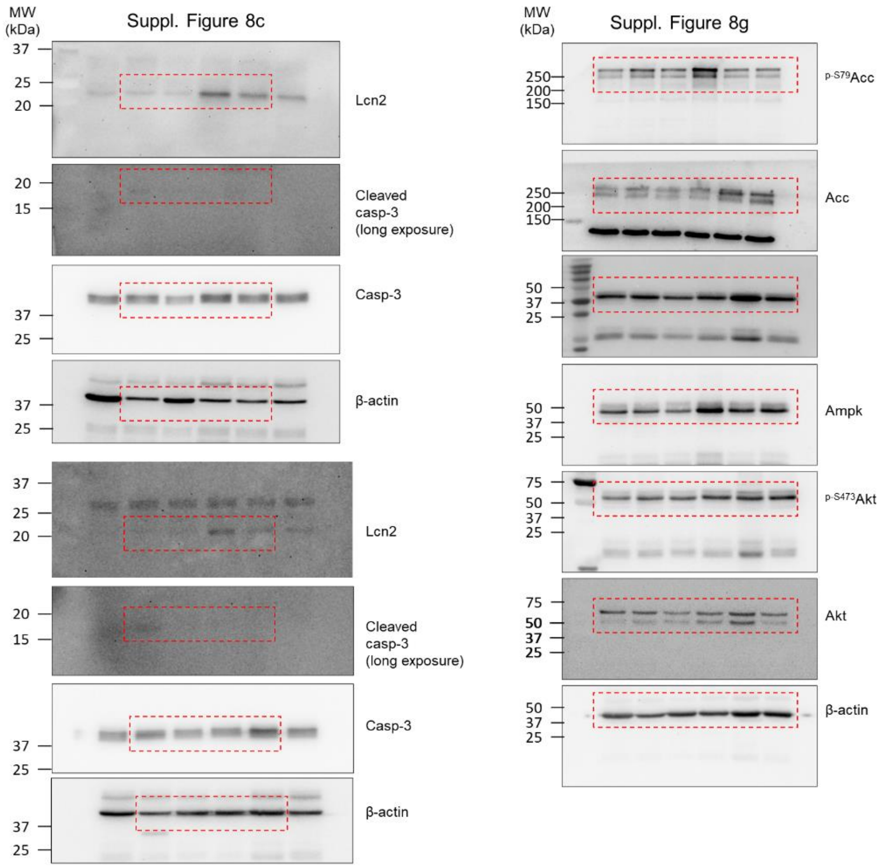

Supplementary Fig. 14. Uncropped and unprocessed versions of western blots presented in the supplementary figures

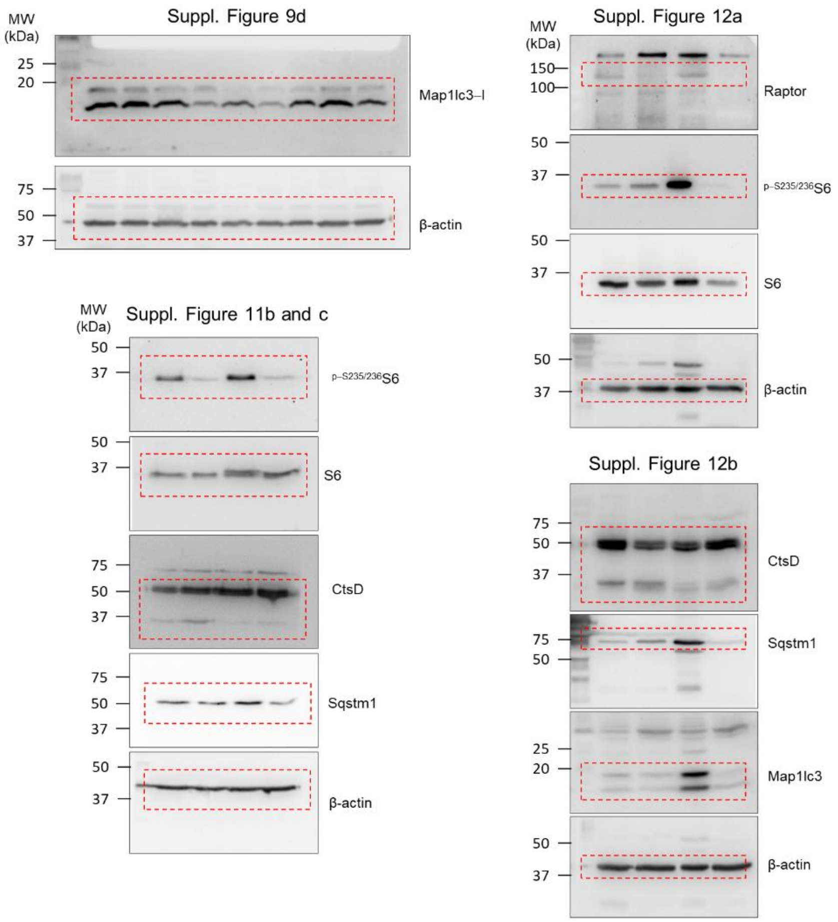

Supplementary Fig. 14. Uncropped and unprocessed versions of western blots presented in the supplementary figures

Supplementary Table 1. Mouse primer pairs used for the gene expression analysis

Gene name	Forward primer (5'-3')	Reverse primer (5'-3')
Gapdh	TGCACCACCAACTGCTTAGC	GGATGCAGGGATGATGTTCT
Cdk1	ACTCCACTCCGGTTGACATC	TCCACTTGGGAAAGGTGTTC
Ccna2	CTTGGCTGCACCAACAGTAA	AGCAATGAGTGAAGGCAGGT
Cdc20	ATGGAGCAGCCTGGAGACTA	GCTTACTCGAGCGGAGTGAC
Ccnb2	TGAAACCAGTGCAGATGGAG	CTGCAGAGCTGAGGGTTCTC
Cdh2	AGGGTGGACGTCATTGTAGC	TGTGACTAGCCCATCATTGC
Tfr	GCGCATTCAAGTGTCTGAAA	GAGCCACAACAGCATGAGAA
F5	TCCCGAGATATTCACGTGGT	TGCCAGCTACCTGGTTTTCT
Apob	CTCTTGCCACAGCTGATTGA	TGAGGGATTTGGGATCAGAG
Lrp2	CAGTGGATTGGGTAGCAG	GCTTGGGGTCAACAACGATA
Tfe3	TCTTCATCACGGGTCTTGCT	TTCTCGAGGTGGGTCTGAAC
Mcoln1	TCCACTTCCAGCTGAAGACA	CTGGATGTGGGTCTTGGTCT
Sqstm1	CCCCAATGTGATCTGTGATG	AAGGGGTTGGGAAAGATGAG
Map1Lc3B	CCGAGAAGACCTTCAAGCAG	CCAGGAACTTGGTCTTCTCC
Lamp1	TAGTGCCCACATTCAGCATCTCCA	TTCCACAGACCCAAACCTGTCACT
CtsD	CCAAGTTTGATGGCATCTTGGGCA	TGGAGTCAGTGCCACCAAGCATTA
Atg9A	CTCCGAGTGATTCTTGCACA	GACGATGGGACTCAGCAACT
Foxm1	AAGGCAAAGACAGGAGAGCT	AGGGCTCCTCAACCTTAACC
Sox9	CAAGAACAAGCCACACGTCA	GTGGTCTTTCTTGTGCTGCA
Vimentin	AATGCTTCTCTGGCACGTCT	AGTGAGGTCAGGCTTGGAAA
Slc7a13	GCCTTTGTTGTTTTGTGCCC	GGGCTCTTGGTACCTCAGTT
Slc5a2	TTGGGCATCACCATGATTTA	GCTCCCAGGTATTTGTCGAA
Slc34a1	GGGAGAAGCTATCCAGCTCA	ACAGCAAACCAGCGGTACTT
Slc3a1	ACTCAGGTGGGAATGCATGA	CGGCTTCCTGGATAAATGGC

Supplementary Table 2. Rat primer pairs used for the gene expression analysis

Gene name	Forward primer (5'-3')	Reverse primer (5'-3')
Slc34a1	GCCCGAGATGTTGAAGAAGA	TCAGTATTGAGCGGGCCTAC
Slc5a2	TCTTTGTGCCCCGTGTTAAT	AGTGTACCCGGCAGAAGATG
*Foxm*1	AAGGCAAAGACAGGAGAGCT	CTGCCAAGACACTGATGCTC
Vimentin	TGCCCTTGAAGCTGCTAACT	AATCCTGCTCTCCCCTT
