# Supplementary material for: Lysosomal cystine export regulates mTORC1 signaling to guide kidney epithelial cell fate specialization
Source: Nat Commun. 2023 Jul 14;14:3994. doi: 10.1038/s41467-023-39261-3 (PMC10349091; doi:10.1038/s41467-023-39261-3)
Supplement: Supplementary file 2 — Reporting Summary [file 41467_2023_39261_MOESM2_ESM.pdf]

nature portfolio

Corresponding author(s): Luciani Alessandro

Last updated by author(s): May 2, 2023

# Reporting Summary

Nature Portfolio wishes to improve the reproducibility of the work that we publish. This form provides structure for consistency and transparency in reporting. For further information on Nature Portfolio policies, see our Editorial Policies and the Editorial Policy Checklist.

## Statistics

For all statistical analyses, confirm that the following items are present in the figure legend, table legend, main text, or Methods section.

| n/a | Confirmed | |
|---|---|---|
| ☐ | ☒ | The exact sample size (*n*) for each experimental group/condition, given as a discrete number and unit of measurement |
| ☐ | ☒ | A statement on whether measurements were taken from distinct samples or whether the same sample was measured repeatedly |
| ☐ | ☒ | The statistical test(s) used AND whether they are one- or two-sided<br>*Only common tests should be described solely by name; describe more complex techniques in the Methods section.* |
| ☒ | ☐ | A description of all covariates tested |
| ☐ | ☒ | A description of any assumptions or corrections, such as tests of normality and adjustment for multiple comparisons |
| ☐ | ☒ | A full description of the statistical parameters including central tendency (e.g. means) or other basic estimates (e.g. regression coefficient) AND variation (e.g. standard deviation) or associated estimates of uncertainty (e.g. confidence intervals) |
| ☐ | ☒ | For null hypothesis testing, the test statistic (e.g. *F*, *t*, *r*) with confidence intervals, effect sizes, degrees of freedom and *P* value noted<br>*Give P values as exact values whenever suitable.* |
| ☒ | ☐ | For Bayesian analysis, information on the choice of priors and Markov chain Monte Carlo settings |
| ☒ | ☐ | For hierarchical and complex designs, identification of the appropriate level for tests and full reporting of outcomes |
| ☐ | ☒ | Estimates of effect sizes (e.g. Cohen's *d*, Pearson's *r*), indicating how they were calculated |

*Our web collection on statistics for biologists contains articles on many of the points above.*

## Software and code

Policy information about availability of computer code

| | |
|---|---|
| Data collection | All tools (Leica SP8 confocal laser scanning microscope, multi-photon fluorescence microscope Leica SP8 MP DIVE Falcon, and ChemiDoc MP) used for collecting data are detailed in the Methods section. |
| Data analysis | All the softwares [Fiji v.2.0.0. (ImageJ, NIH), Huygens (Scientific Volume Imaging, Hilversum, The Netherlands), Ilastik (EMBL, Heideberg, Germany), NetworkAnalyst, MeboAnalyst, OmicsNet2.0, CellProfiler, GraphPad v.9.4.1] used for data analysis are detailed in the Methods section. |

For manuscripts utilizing custom algorithms or software that are central to the research but not yet described in published literature, software must be made available to editors and reviewers. We strongly encourage code deposition in a community repository (e.g. GitHub). See the Nature Portfolio guidelines for submitting code & software for further information.

## Data

Policy information about availability of data

All manuscripts must include a data availability statement. This statement should provide the following information, where applicable:

- Accession codes, unique identifiers, or web links for publicly available datasets
- A description of any restrictions on data availability
- For clinical datasets or third party data, please ensure that the statement adheres to our policy

The RNA sequencing and microarray data generated in this study have been deposited to the NCBI's Gene Expression Omnibus (GEO) under accession codes

GSE226397 (RNA seq) and GSE226396 (Microarray), respectively. The mass spectrometry-based proteomics data have been deposited to ProteomeXchange Consortium with the identifier code PXD040450. The mass-spectrometry-based metabolomics data have been deposited to ZENODO with the identifier code (105281/zenodo.765521). Source data are provided with this paper. All data supporting the findings of this study are available from the corresponding authors on reasonable request.

## Human research participants

Policy information about studies involving human research participants and Sex and Gender in Research.

| | |
|---|---|
| Reporting on sex and gender | N/A |
| Population characteristics | N/A |
| Recruitment | N/A |
| Ethics oversight | N/A |

Note that full information on the approval of the study protocol must also be provided in the manuscript.

# Field-specific reporting

Please select the one below that is the best fit for your research. If you are not sure, read the appropriate sections before making your selection.

☒ Life sciences ☐ Behavioural & social sciences ☐ Ecological, evolutionary & environmental sciences

For a reference copy of the document with all sections, see nature.com/documents/nr-reporting-summary-flat.pdf

# Life sciences study design

All studies must disclose on these points even when the disclosure is negative.

| | |
|---|---|
| Sample size | No statistical methods were used to predetermine the sample size. The sample size was selected based on commonly adopted standards in the field, resulting in statistically meaningful comparisons. |
| Data exclusions | No data were excluded from the analysis. |
| Replication | All of the experiments reported here in this study were independently performed two to three times or more as indicated in the legends. |
| Randomization | No randomization was applied in this study. |
| Blinding | The investigators were not blinded to allocation during the experiments and outcome assessment. |

# Behavioural & social sciences study design

All studies must disclose on these points even when the disclosure is negative.

| | |
|---|---|
| Study description | *Briefly describe the study type including whether data are quantitative, qualitative, or mixed-methods (e.g. qualitative cross-sectional, quantitative experimental, mixed-methods case study).* |
| Research sample | *State the research sample (e.g. Harvard university undergraduates, villagers in rural India) and provide relevant demographic information (e.g. age, sex) and indicate whether the sample is representative. Provide a rationale for the study sample chosen. For studies involving existing datasets, please describe the dataset and source.* |
| Sampling strategy | *Describe the sampling procedure (e.g. random, snowball, stratified, convenience). Describe the statistical methods that were used to predetermine sample size OR if no sample-size calculation was performed, describe how sample sizes were chosen and provide a rationale for why these sample sizes are sufficient. For qualitative data, please indicate whether data saturation was considered, and what criteria were used to decide that no further sampling was needed.* |
| Data collection | *Provide details about the data collection procedure, including the instruments or devices used to record the data (e.g. pen and paper, computer, eye tracker, video or audio equipment) whether anyone was present besides the participant(s) and the researcher, and whether the researcher was blind to experimental condition and/or the study hypothesis during data collection.* |
| Timing | *Indicate the start and stop dates of data collection. If there is a gap between collection periods, state the dates for each sample cohort.* |

| | |
|---|---|
| Data exclusions | *If no data were excluded from the analyses, state so OR if data were excluded, provide the exact number of exclusions and the rationale behind them, indicating whether exclusion criteria were pre-established.* |
| Non-participation | *State how many participants dropped out/declined participation and the reason(s) given OR provide response rate OR state that no participants dropped out/declined participation.* |
| Randomization | *If participants were not allocated into experimental groups, state so OR describe how participants were allocated to groups, and if allocation was not random, describe how covariates were controlled.* |

# Ecological, evolutionary & environmental sciences study design

All studies must disclose on these points even when the disclosure is negative.

| | |
|---|---|
| Study description | *Briefly describe the study. For quantitative data include treatment factors and interactions, design structure (e.g. factorial, nested, hierarchical), nature and number of experimental units and replicates.* |
| Research sample | *Describe the research sample (e.g. a group of tagged Passer domesticus, all Stenocereus thurberi within Organ Pipe Cactus National Monument), and provide a rationale for the sample choice. When relevant, describe the organism taxa, source, sex, age range and any manipulations. State what population the sample is meant to represent when applicable. For studies involving existing datasets, describe the data and its source.* |
| Sampling strategy | *Note the sampling procedure. Describe the statistical methods that were used to predetermine sample size OR if no sample-size calculation was performed, describe how sample sizes were chosen and provide a rationale for why these sample sizes are sufficient.* |
| Data collection | *Describe the data collection procedure, including who recorded the data and how.* |
| Timing and spatial scale | *Indicate the start and stop dates of data collection, noting the frequency and periodicity of sampling and providing a rationale for these choices. If there is a gap between collection periods, state the dates for each sample cohort. Specify the spatial scale from which the data are taken* |
| Data exclusions | *If no data were excluded from the analyses, state so OR if data were excluded, describe the exclusions and the rationale behind them, indicating whether exclusion criteria were pre-established.* |
| Reproducibility | *Describe the measures taken to verify the reproducibility of experimental findings. For each experiment, note whether any attempts to repeat the experiment failed OR state that all attempts to repeat the experiment were successful.* |
| Randomization | *Describe how samples/organisms/participants were allocated into groups. If allocation was not random, describe how covariates were controlled. If this is not relevant to your study, explain why.* |
| Blinding | *Describe the extent of blinding used during data acquisition and analysis. If blinding was not possible, describe why OR explain why blinding was not relevant to your study.* |

Did the study involve field work? ☐ Yes ☐ No

## Field work, collection and transport

| | |
|---|---|
| Field conditions | *Describe the study conditions for field work, providing relevant parameters (e.g. temperature, rainfall).* |
| Location | *State the location of the sampling or experiment, providing relevant parameters (e.g. latitude and longitude, elevation, water depth).* |
| Access & import/export | *Describe the efforts you have made to access habitats and to collect and import/export your samples in a responsible manner and in compliance with local, national and international laws, noting any permits that were obtained (give the name of the issuing authority, the date of issue, and any identifying information).* |
| Disturbance | *Describe any disturbance caused by the study and how it was minimized.* |

# Reporting for specific materials, systems and methods

We require information from authors about some types of materials, experimental systems and methods used in many studies. Here, indicate whether each material, system or method listed is relevant to your study. If you are not sure if a list item applies to your research, read the appropriate section before selecting a response.

## Materials & experimental systems

| n/a | Involved in the study |
| --- | --- |
| [ ] | [X] Antibodies |
| [ ] | [X] Eukaryotic cell lines |
| [ ] | [ ] Palaeontology and archaeology |
| [ ] | [X] Animals and other organisms |
| [X] | [ ] Clinical data |
| [X] | [ ] Dual use research of concern |

## Methods

| n/a | Involved in the study |
| --- | --- |
| [X] | [ ] ChIP-seq |
| [X] | [ ] Flow cytometry |
| [X] | [ ] MRI-based neuroimaging |

# Antibodies

Antibodies used

The following antibodies were used in this study: anti-LAMP1 (Santa Cruz Biotechnology, sc-19992, 1:1000); anti-LC3 (PM036, MBL, 1:200); anti-p62/SQSTM1 (PM045, MBL, 1:200); anti-phospho-S6 Ribosomal Protein (Ser235/236; Cell signaling technology, 4858, 1:500), anti-cathepsin D (Santa Cruz Biotechnology, sc-6486, 1:500), anti-S6 Ribosomal Protein (Cell Signaling Technology, 2217, 1:500), anti-phospho–4E-BP1 (Ser65; Cell signaling technology, 9451, 1:500), anti-4E-BP1 (Cell Signaling Technology, 9644, 1:500), anti-phospho-p70 S6 kinase 1 (Thr389; Cell Signaling Technology, 9234, 1:500); anti-p70 S6 kinase 1 (Cell Signaling Technology, 2708, 1:500), anti-acetyl–CoA carboxylase (Cell signaling, 3662, 1:500); anti-phospho–acetyl–CoA carboxylase (Ser79; Cell signaling, 3661, 1:500); anti-AMPKα (Cell signaling, 2793, 1:500); anti-phospho–AMPKα (Thr172; Cell signaling, 2535, 1:500), anti-TFE3 (Sigma, HPA023881, 1:500); anti-mTOR (Cell Signaling Technology, 7C10, 1:400 for immunofluorescence studies), anti-Raptor (2280, Cell Signaling Technology, 2280, 1:400); anti�RagC (Cell Signaling Technology, 5466, 1:500); rabbit anti-phospho-AKT (Ser473; Cell Signaling Technology, 13038, 1:500), anti-AQP1(Aviva Systems Biology, OASA00210, 1:400), anti-PCNA (Dako, M0879, 1:500), anti-α-tubulin (Sigma Aldrich, T5168; 1:1000), anti-Cyclin A2 (Abcam, Ab181591; 1:400), anti Cdc20 (Santa Cruz, sc-13162, 1:400), anti-Golgin-97 (Cell Signaling Technology, 13192; 1:400); anti-VDAC (Cell Signaling Technology; 4866, 1:400), anti-PMP70 (Sigma, SAB4200181, 1:500), anti-calreticulin (Cell Signaling Technology, 12238, 1:400), anti-β-actin (Sigma-Aldrich, A5441, 1:1000), anti-�-tubulin (Sigma Aldrich, clone GTU-88,1:10000), and anti-HA (Roche, 11867423001; 1:500), anti-acetylated-tubulin (Sigma,T7451; 1:400), Anti-Laminin gamma 1 (clone A5; Thermo Fisher Scientific, MA106100, 1:400), Atp6v0d1 (Thermo Fisher Scientific, PA5-103179, 1:200) ATP6V1B2 (D2F9R, Cell signaling,14617, 1:200), anti-vimentin (Abcam, Ab92547, 1:400), anti-Lipoclain2/NGAL (R&D systems, AF1857, 1:400), anti-cleaved caspase 3 (Cell signaling), 9662, 1:400), anti-caspase 3 (Cell signaling, 9661, 1:400), anti-megalin (1:1000) was kindly provided by P. Verroust and R. Kozyraki (INSERM, Paris, France), donkey-anti rabbit IgG, cross-absorbed secondary antibody, Alexa Fluor 488 (Thermo Fischer Scientific, A10040, 1:400), donkey-anti mouse IgG, cross-absorbed secondary antibody, Alexa Fluor 488 (Thermo Fischer Scientific, A21202, 1:400), donkey-anti rat IgG, cross-absorbed secondary antibody, Alexa Fluor 488 (Thermo Fischer Scientific, A21202, 1:400), , donkey-anti rat IgG, cross-absorbed secondary antibody, Alexa Fluor 647 (Thermo Fischer Scientific, A31573, 1:400), donkey-anti goat IgG, cross-absorbed secondary antibody, Alexa Fluor 633 (Thermo Fischer Scientific, A21094, 1:400), donkey-anti goat IgG, cross-absorbed secondary antibody, Alexa Fluor 546 (Thermo Fischer Scientific, A11056, 1:400), donkey-anti rat IgG, cross-absorbed secondary antibody, Alexa Fluor 546 (Thermo Fischer Scientific, A11081, 1:400).

Validation

All antibodies used in our study have been previously characterized and validated by biological strategies. Detailed information can be found on the website from manufactures. This information has been listed in the methods section.

# Eukaryotic cell lines

Policy information about cell lines and Sex and Gender in Research

Cell line source(s)

Primary cultures of micro-dissected proximal tubules derived from mouse kidneys were developed in the Devuyst lab at Institute of Physiology, University of Zurich.

Authentication

Primary proximal tubule cells were authenticated by their gene profile and by assessing their specialized functions such as endocytic uptake of albumin and transferrin ligands.

Mycoplasma contamination

All cell lines were routinely tested for mycoplasma using a commercial kit (Lonza) and verified as mycoplasma-negative.

Commonly misidentified lines
(See ICLAC register)

N/A

# Palaeontology and Archaeology

Specimen provenance

*Provide provenance information for specimens and describe permits that were obtained for the work (including the name of the issuing authority, the date of issue, and any identifying information). Permits should encompass collection and, where applicable, export.*

Specimen deposition

*Indicate where the specimens have been deposited to permit free access by other researchers.*

Dating methods

*If new dates are provided, describe how they were obtained (e.g. collection, storage, sample pretreatment and measurement), where they were obtained (i.e. lab name), the calibration program and the protocol for quality assurance OR state that no new dates are provided.*

☐ Tick this box to confirm that the raw and calibrated dates are available in the paper or in Supplementary Information.

Ethics oversight

*Identify the organization(s) that approved or provided guidance on the study protocol, OR state that no ethical approval or guidance was required and explain why not.*

Note that full information on the approval of the study protocol must also be provided in the manuscript.

## Animals and other research organisms

Policy information about studies involving animals; ARRIVE guidelines recommended for reporting animal research, and Sex and Gender in Research

| | |
|---|---|
| Laboratory animals | Experiments were conducted on age- and gender-matched Ctns knockout mouse (C57BL/6 background4) or rat (Sprague-Dawley background) lines, and their corresponding control littermates. Due to an effect of sex on cystine content (higher levels in female versus male kidneys), only female mice at age 6, 12, and 24 weeks were used in all experiments and cellular studies. Rats and mice were housed under specific pathogen free conditions and maintained under temperature (22-25°C)-and humidity (50-60%)-controlled conditions with 12-h dark/light cycle with water and food provided ad libitum. Zebrafish larvae (AB background) were kept at day/night cycle of 14/10 h at 28°C and fed with XX and solid fish food XX starting at 5 d.p.f. Most of the experiments were performed on larvae at 5-14 d.p.f. Animal sex is not specified at this developmental stage. |
| Wild animals | No wild animals were involved in this study. |
| Reporting on sex | Gender was considered only for the mouse model, as there has been described a sex-dependent effect on cystine storage and hence kidney disease development in females versus males. No sex-based analyses in rats. |
| Field-collected samples | This study did not involve samples collected from the field. |
| Ethics oversight | All the animal experiments were approved by the Cantonal Veterinary Office Zurich (license no. ZH195/2020, ZH139/2022, and ZH230/2019) and performed in accordance with the guidelines of the University of Zurich. |

Note that full information on the approval of the study protocol must also be provided in the manuscript.

## Clinical data

Policy information about clinical studies

All manuscripts should comply with the ICMJE guidelines for publication of clinical research and a completed CONSORT checklist must be included with all submissions.

| | |
|---|---|
| Clinical trial registration | *Provide the trial registration number from ClinicalTrials.gov or an equivalent agency.* |
| Study protocol | *Note where the full trial protocol can be accessed OR if not available, explain why.* |
| Data collection | *Describe the settings and locales of data collection, noting the time periods of recruitment and data collection.* |
| Outcomes | *Describe how you pre-defined primary and secondary outcome measures and how you assessed these measures.* |

## Dual use research of concern

Policy information about dual use research of concern

### Hazards

Could the accidental, deliberate or reckless misuse of agents or technologies generated in the work, or the application of information presented in the manuscript, pose a threat to:

| No | Yes | |
|---|---|---|
| ☒ | ☐ | Public health |
| ☒ | ☐ | National security |
| ☒ | ☐ | Crops and/or livestock |
| ☒ | ☐ | Ecosystems |
| ☒ | ☐ | Any other significant area |

## Experiments of concern

Does the work involve any of these experiments of concern:

| No | Yes | |
|---|---|---|
| ☒ | ☐ | Demonstrate how to render a vaccine ineffective |
| ☒ | ☐ | Confer resistance to therapeutically useful antibiotics or antiviral agents |
| ☒ | ☐ | Enhance the virulence of a pathogen or render a nonpathogen virulent |
| ☒ | ☐ | Increase transmissibility of a pathogen |
| ☒ | ☐ | Alter the host range of a pathogen |
| ☒ | ☐ | Enable evasion of diagnostic/detection modalities |
| ☒ | ☐ | Enable the weaponization of a biological agent or toxin |
| ☒ | ☐ | Any other potentially harmful combination of experiments and agents |

# ChIP-seq

## Data deposition

☐ Confirm that both raw and final processed data have been deposited in a public database such as GEO.

☐ Confirm that you have deposited or provided access to graph files (e.g. BED files) for the called peaks.

| | |
|---|---|
| Data access links<br>*May remain private before publication.* | *For "Initial submission" or "Revised version" documents, provide reviewer access links. For your "Final submission" document, provide a link to the deposited data.* |
| Files in database submission | *Provide a list of all files available in the database submission.* |
| Genome browser session<br>(e.g. UCSC) | *Provide a link to an anonymized genome browser session for "Initial submission" and "Revised version" documents only, to enable peer review. Write "no longer applicable" for "Final submission" documents.* |

## Methodology

| | |
|---|---|
| Replicates | *Describe the experimental replicates, specifying number, type and replicate agreement.* |
| Sequencing depth | *Describe the sequencing depth for each experiment, providing the total number of reads, uniquely mapped reads, length of reads and whether they were paired- or single-end.* |
| Antibodies | *Describe the antibodies used for the ChIP-seq experiments; as applicable, provide supplier name, catalog number, clone name, and lot number.* |
| Peak calling parameters | *Specify the command line program and parameters used for read mapping and peak calling, including the ChIP, control and index files used.* |
| Data quality | *Describe the methods used to ensure data quality in full detail, including how many peaks are at FDR 5% and above 5-fold enrichment.* |
| Software | *Describe the software used to collect and analyze the ChIP-seq data. For custom code that has been deposited into a community repository, provide accession details.* |

# Flow Cytometry

## Plots

Confirm that:

☐ The axis labels state the marker and fluorochrome used (e.g. CD4-FITC).

☐ The axis scales are clearly visible. Include numbers along axes only for bottom left plot of group (a 'group' is an analysis of identical markers).

☐ All plots are contour plots with outliers or pseudocolor plots.

☐ A numerical value for number of cells or percentage (with statistics) is provided.

## Methodology

| | |
|---|---|
| Sample preparation | *Describe the sample preparation, detailing the biological source of the cells and any tissue processing steps used.* |
| Instrument | *Identify the instrument used for data collection, specifying make and model number.* |

Software | *Describe the software used to collect and analyze the flow cytometry data. For custom code that has been deposited into a community repository, provide accession details.*

Cell population abundance | *Describe the abundance of the relevant cell populations within post-sort fractions, providing details on the purity of the samples and how it was determined.*

Gating strategy | *Describe the gating strategy used for all relevant experiments, specifying the preliminary FSC/SSC gates of the starting cell population, indicating where boundaries between "positive" and "negative" staining cell populations are defined.*

☐ Tick this box to confirm that a figure exemplifying the gating strategy is provided in the Supplementary Information.

# Magnetic resonance imaging

## Experimental design

Design type | *Indicate task or resting state; event-related or block design.*

Design specifications | *Specify the number of blocks, trials or experimental units per session and/or subject, and specify the length of each trial or block (if trials are blocked) and interval between trials.*

Behavioral performance measures | *State number and/or type of variables recorded (e.g. correct button press, response time) and what statistics were used to establish that the subjects were performing the task as expected (e.g. mean, range, and/or standard deviation across subjects).*

## Acquisition

Imaging type(s) | *Specify: functional, structural, diffusion, perfusion.*

Field strength | *Specify in Tesla*

Sequence & imaging parameters | *Specify the pulse sequence type (gradient echo, spin echo, etc.), imaging type (EPI, spiral, etc.), field of view, matrix size, slice thickness, orientation and TE/TR/flip angle.*

Area of acquisition | *State whether a whole brain scan was used OR define the area of acquisition, describing how the region was determined.*

Diffusion MRI ☐ Used ☐ Not used

## Preprocessing

Preprocessing software | *Provide detail on software version and revision number and on specific parameters (model/functions, brain extraction, segmentation, smoothing kernel size, etc.).*

Normalization | *If data were normalized/standardized, describe the approach(es): specify linear or non-linear and define image types used for transformation OR indicate that data were not normalized and explain rationale for lack of normalization.*

Normalization template | *Describe the template used for normalization/transformation, specifying subject space or group standardized space (e.g. original Talairach, MNI305, ICBM152) OR indicate that the data were not normalized.*

Noise and artifact removal | *Describe your procedure(s) for artifact and structured noise removal, specifying motion parameters, tissue signals and physiological signals (heart rate, respiration).*

Volume censoring | *Define your software and/or method and criteria for volume censoring, and state the extent of such censoring.*

## Statistical modeling & inference

Model type and settings | *Specify type (mass univariate, multivariate, RSA, predictive, etc.) and describe essential details of the model at the first and second levels (e.g. fixed, random or mixed effects; drift or auto-correlation).*

Effect(s) tested | *Define precise effect in terms of the task or stimulus conditions instead of psychological concepts and indicate whether ANOVA or factorial designs were used.*

Specify type of analysis: ☐ Whole brain ☐ ROI-based ☐ Both

Statistic type for inference (See Eklund et al. 2016) | *Specify voxel-wise or cluster-wise and report all relevant parameters for cluster-wise methods.*

Correction | *Describe the type of correction and how it is obtained for multiple comparisons (e.g. FWE, FDR, permutation or Monte Carlo).*

## Models & analysis

| n/a | Involved in the study |
|---|---|
| ☒ | ☐ Functional and/or effective connectivity |
| ☒ | ☐ Graph analysis |
| ☒ | ☐ Multivariate modeling or predictive analysis |
